# Transcriptional rewiring in CD8^+^ T cells: implications for CAR-T cell therapy against solid tumours

**DOI:** 10.3389/fimmu.2024.1412731

**Published:** 2024-09-27

**Authors:** Shamini Srinivasan, Jesse Armitage, Jonas Nilsson, Jason Waithman

**Affiliations:** ^1^ School of Biomedical Sciences, The University of Western Australia, Perth, WA, Australia; ^2^ Telethon Kids Cancer Centre, Telethon Kids Institute, Perth, WA, Australia; ^3^ Melanoma Discovery Lab, Harry Perkins Institute of Medical Research, Centre of Medical Research, The University of Western Australia, Perth, WA, Australia; ^4^ Sahlgrenska Center for Cancer Research, Department of Surgery, Institute of Clinical Sciences, University of Gothenburg, Gothenburg, Sweden

**Keywords:** immunotherapy, adoptive cell therapy (ACT), transcription factor, cancer, CAR-T cell, CD8^+^ T cell, solid tumour

## Abstract

T cells engineered to express chimeric-antigen receptors (CAR-T cells) can effectively control relapsed and refractory haematological malignancies in the clinic. However, the successes of CAR-T cell therapy have not been recapitulated in solid tumours due to a range of barriers such as immunosuppression, poor infiltration, and tumour heterogeneity. Numerous strategies are being developed to overcome these barriers, which include improving culture conditions and manufacturing protocols, implementing novel CAR designs, and novel approaches to engineering the T cell phenotype. In this review, we describe the various emerging strategies to improve CAR T cell therapy for solid tumours. We specifically focus on new strategies to modulate cell function and fate that have precipitated from the growing knowledge of transcriptional circuits driving T cell differentiation, with the ultimate goal of driving more productive anti-tumour T cell immunity. Evidence shows that enrichment of particular phenotypic subsets of T cells in the initial cell product correlates to improved therapeutic responses and clinical outcomes. Furthermore, T cell exhaustion and poor persistence are major factors limiting therapeutic efficacy. The latest preclinical work shows that targeting specific master regulators and transcription factors can overcome these key barriers, resulting in superior T cell therapeutic products. This can be achieved by targeting key transcriptional circuits promoting memory-like phenotypes or sustaining key effector functions within the hostile tumour microenvironment. Additional discussion points include emerging considerations for the field such as (i) targeting permutations of transcription factors, (ii) transient expression systems, (iii) tissue specificity, and (iv) expanding this strategy beyond CAR-T cell therapy and cancer.

## 1 Introduction

A critical arm of cancer immunity involves CD8^+^ T cell activity. CD8^+^ T cells are cytotoxic lymphocytes that eliminate infected and malignant cells based on the recognition of non-self or altered-self antigens presented by class I major histocompatibility complex (MHC) molecules on the cell surface. Recent decades of basic, fundamental research have seen rapid growth in our understanding of T cell biology, from the stringent selection criteria enforced during T cell development to their striking phenotypic plasticity post activation. This provided the foundations for driving important discovery research down the translational pipeline, leading to a critical new pillar of treatment known as cancer immunotherapy. This treatment modality has drawn immense scientific and clinical attention due to its ability to drastically improve outcomes for aggressive cancers through two primary streams: (*i*) immune checkpoint blockade (ICB) and (*ii*) adoptive cell therapy (ACT) (1). ICB has achieved impressive clinical outcomes for patients with advanced solid tumours, and include CTLA-4 inhibitors (e.g. ipilimumab), PD-1/PD-L1 inhibitors (e.g. nivolumab, pembrolizumab and atezolimumab) and LAG-3 inhibitors (e.g. relatlimab) (2). ACT protocols employ either tumour-infiltrating lymphocytes (TILs) grown out from tumours in the presence of interleukin-2, or chimeric antigen receptor (CAR)-T cells, which are genetically engineered T cells expressing a synthetic receptor that redirects cytotoxicity towards target antigens. TIL therapy consists of an autologous T cell product that has shown efficacy in a few solid tumours (3). CAR-T cells have demonstrated remarkable remissions achieved for patients with various haematological cancers (4). Very recently, a TCR transgenic T cell product was approved by the FDA for the rare but aggressive synovial subtype of sarcoma (5). However, despite these clinical successes, many patients present with immunotherapy-resistant disease, often due to failures associated with the CD8^+^ T cell response. Thus, defining the molecular drivers and mechanisms underpinning highly effective CD8^+^ T cell immunity remains an important goal in the field for improving therapeutic outcomes.

The activated CD8^+^ T cell compartment is remarkably diverse. In acute infections, CD8^+^ T cells adopt a continuum of functional states, from short-lived effector cells that efficiently eliminate infected cells, to quiescent memory populations that provide long-lived immunity for the host (6). More recently, it has been shown that persistent antigen exposure, a condition frequently observed with chronic infection or cancer, generates an additional spectrum of ‘exhausted’ phenotypes, varying in functional capacities and stem-like features (7). The formation of these different phenotypes are directed by an array of extracellular signals, such as the strength of antigen signalling, the cytokine milieu, cell-to-cell communication, and the availability of metabolites and nutrients. Transcription factors play a crucial role in establishing these heterogeneous cellular phenotypes, and as such, play a central role in shaping CD8^+^ T cell activity and maintenance. Importantly, the balance of these different phenotypes dictates the quality of CD8^+^ T cell responses, including the efficacy of therapeutic products targeting this immune compartment.

Transcription factors are proteins that bind to key regulatory elements in the DNA to regulate gene expression and steer the developmental trajectory of cells. Transcriptional networks guide the developmental program of cells over our lifetime, regulating the differentiation of embryonic stem cells into the numerous unique cell types found throughout the body. A striking demonstration of the control that transcription factors have on cellular fate is the ability of forced expression of the OKSM/Yamanaka factors (four transcription factors: Oct3/4, Sox2, Klf4, and c-Myc) to reprogram adult human and mouse fibroblasts back into pluripotent stem cells (8, 9). Due to the advent of novel genetic engineering platforms, bioinformatics approaches, and high throughput screening tools, we are on an exciting precipice of unravelling the numerous transcriptional networks underpinning effective CD8^+^ T cell. It has become evident that the transcription factors regulating these networks serve as actionable molecular targets that can be leveraged to enhance the performance of cellular therapies. Thus, there is significant interest in identifying and modulating the expression of transcription factors in CD8^+^ T cells to improve ACT protocols, with a particular desire to extend its clinical application into the realm of treatment-resistant solid tumours. This review aims to provide an up-to-date view of transcription factors that have been targeted in preclinical T cell therapy protocols, and the outcomes of this strategy. We discuss novel approaches to deciphering and rewiring these transcriptional circuits, such as using revolutionary next-generation sequencing technologies and emerging genetic engineering platforms and their applications to cancer immunotherapy. Finally, in light of this cutting-edge research, we speculate on the future directions of this field.

## 2 T cell development and differentiation

A T cell’s journey begins with the migration of common lymphoid progenitors from the bone-marrow to the thymus. It is here that thymocytes are programmed and selected to develop into naive CD4^+^ or CD8^+^ T cells, steered towards their fate by lineage-determining transcription factors (10, 11). Random recombination of T cell receptor (TCR) genes generates an extraordinarily diverse repertoire of antigen-specificities, which is pruned by selective processes to calibrate T cell responses towards ‘altered self’. Once selection is complete, successful naive T cells undergo a small burst of proliferation, followed by each naïve clone emerging from the thymus to seed the periphery. These naive T cells are destined to circulate throughout the secondary lymphoid compartment, traversing the host’s intricate immune surveillance network in search of their cognate antigen. During this stage they are transcriptionally and epigenetically maintained in a state of quiescence and homeostasis (12). Whilst many naive T cells never undergo activation, those that encounter their cognate antigen on an antigen presenting cell (APC) undergo extensive transcriptional, epigenetic, and metabolic rewiring to initiate rapid clonal expansion. T cell activation requires three signals: antigen recognition provides the first signal, costimulation provides the second signal, and cytokine support provides the third signal. This results in the generation of a highly specific army of T cells directed against infected or malignant cells.

The formation of these different phenotypes are directed by an array of extracellular signals, such as the strength of antigen signalling, the cytokine milieu, cell-to-cell communication, and the availability of metabolites and nutrients. Transcription factors play a crucial role in establishing these heterogeneous cellular phenotypes, and as such, play a central role in shaping CD8^+^ T cell activity and maintenance. Importantly, the balance of these different phenotypes dictates the quality of CD8^+^ T cell responses, including the efficacy of therapeutic products targeting this immune compartment.

## 3 CAR-T cell therapy in solid tumours

ACT is an established, powerful branch of immunotherapy that broadly refers to *ex vivo* expansion and modulation of autologous T lymphocytes that are re-infused into patients as a ‘living drug’ to directly target cancer cells. Whilst cancer vaccines and ICB rely on the stimulation and reinvigoration of natural immunity, ACT bypasses the need to promote clonal expansion *in vivo* by expanding lymphocyte numbers in the laboratory (13). CAR-T cells are one form of ACT where T cells are engineered to express synthetic receptors constructed by fusing a surface antigen binding domain to T cell receptor and co-receptor signalling domains, redirecting T cell specificity towards target surface antigens. To date, the impact of CAR-T cell therapies has expanded across a range of haematologic malignancies, primarily targeting CD19 and CD20 on B cell-derived cancers, and B cell maturation antigen (BCMA) on multiple myeloma (**Table 1**).

**Table 1:** Approval of CAR-T cell therapies for various haematological malignancies across the globe, their design (target antigen and costimulatory domain) and key clinical trials investigating the clinical efficacy for respective clinical indications.

Approved CAR-T cell therapies and related trials										
Generic Name and trade name(s)	CAR Design	Clinical Indication	Region Approved				Pivotal Clinical Trials			
			US	EU	JP	AU	Trial Name	Primary endpoint	Median follow-up/cut-off (months)	Outcome
**Tisagenleucel** Kymriah, CTL019, CART-19	Anti-CD19, 4-1BB	R/R B cell ALL	•	•	•	•	**ELIANA** (244)	ORR	3	81%
		R/R FL	•	•			**ELARA** (245)	CRR	16.59	69.1%
		R/R DLBCL	•	•	•	•	**JULIET** (246)	BORR	14.0	52%
**Axicabtagene ciloleucel** Yescarta, Axi-cel, KTE-C19	Anti-CD19, 4-1BB	R/R LBCL	•	•	•	•	**ZUMA-7** (247)	EFS	24.9	8.3 months
		R/R FL	•	•			**ZUMA-5** (248)	ORR	17.5	92%
**Brexucabtagene autoleucel** Tescartus, KTE-X19	Anti-CD19, CD28	R/R B cell ALL	•	•			**ZUMA-3** (249)	CRR	16.4	71%
		R/R MCL	•	•		•	**ZUMA-2** (250)	ORR	12.3	93%
**Lisocabtagene maraleucel** Breyanzi, JCAR017, Liso-cel	Anti-CD19, 4-1BB	R/R LBCL	•	•	•		**TRANSCEND NHL 001** (251)	ORR	18.8	73%
		R/R PMBCL	•	•	•					
		R/R FL	•	•	•					
**Idecabtagene vileucel** Abecma, Ide-cel	Anti-BCMA, 4-1BB	R/R MM	•	•	•		**KarMMa** (252)	ORR	13.3	73%
**Ciltacabtagene autoleucel** Carvykti, Cilta-cel	Anti-BCMA, 4-1BB	R/R MM	•	•	•		**CARTITUDE-1** (253)	ORR	12.4	91%

R/R, relapsed or refractory; ALL, acute lymphoblastic leukaemia; FL, follicular lymphoma; DLBCL, diffuse large B cell lymphoma; LBCL, large B cell lymphoma; MCL, mantle cell lymphoma, PMBCL, primary mediastinal large B cell lymphoma; MM, multiple myeloma; US, United States of America; EU, Europe; JP, Japan; AU, Australia; ORR, objective response rate; CRR, complete response rate; BORR, best overall response rate; EFS, event free survival.

Although CAR-T cell therapy has undoubtedly saved many lives, clinical responses to CAR-T cell therapy are varied, with some patients experiencing no responses or relapses post treatment. Possible reasons for this include poor quality of the initial T cells, poor expansion of CAR-T cells, and poor persistence of the cells over time. Furthermore, although CAR-T cell therapy has improved therapeutic outcomes for subsets of patients with haematological cancers, there remains a clear unmet clinical need for better treatments for patients with advanced solid tumours (14). In contrast to haematological ‘liquid’ tumours, solid tumours are composed of highly dynamic and complex ecosystems commonly referred to as the tumour microenvironment (TME). Clinical efficacy is hindered by several challenges intrinsically present within the TME, including tumour heterogeneity, dysregulated trafficking and infiltration, T cell exhaustion and immunosuppression (15). Heterogeneous antigen expression too often leads to incomplete eradication of tumours by promoting the outgrowth of antigen-negative clones or loss-of antigen variants. Approaches to overcome this include the development of CAR-T cells targeting multiple antigens, such as the ‘quad’ CAR-T cells in the Brainchild-04 Phase I clinical trial (16), or boosting epitope spreading by enhancing DC cross presentation and recruitment of endogenous T cell responses using cytokines such as FLT3L (17) and IL-12 (18). CAR-T cells must also effectively home to the tumour site and successfully infiltrate the tumour. Features of the TME such as abnormal vasculature, obstructive extracellular matrix, and dysregulated chemokine signalling inhibit effective trafficking and infiltration of CAR-T cells. To enhance trafficking and penetrance, studies have investigated genetically engineered expression of chemokine receptors (19), local and/or regional cell delivery (20), and administration of anti-VEGF drugs to stabilise aberrant vasculature (21). Furthermore, prolonged periods of antigen exposure within the TME drives T cells towards exhaustion. T cell function is further dampened by expression of inhibitory molecules on cancer cells, and additional microenvironmental factors such as hypoxia, limited metabolites, and co-opted immunosuppressive cells. Different approaches such as dominant negative receptors (22), knock-out of inhibitory receptors (23), and modified manufacturing protocols with the inclusion of different cytokines or molecules (24, 25) have been studied to enhance resistance to exhaustion and immunosuppression. Providing cytokine support can additionally overcome poor persistence of T cells, a key barrier for therapeutic efficacy in both haematological and solid tumours. Indeed, studies in mice have demonstrated that the survival and therapeutic efficacy of anti-HER2 CAR-T cells is dramatically improved in mice transgenic for interleukin (IL)-2 (26, 27). With the numerous challenges of effectively targeting solid tumours with CAR-T cell therapy, it is beneficial to develop novel strategies that can augment multiple aspects of the CAR-T cell. One such strategy is modulating the expression of transcription factors, master regulators of cell function and fate, to enhance the CAR-T cell phenotype and address multiple barriers to therapeutic efficacy.

## 4 Using transcription factors to rewire CAR-T cells

**Figure 1:**
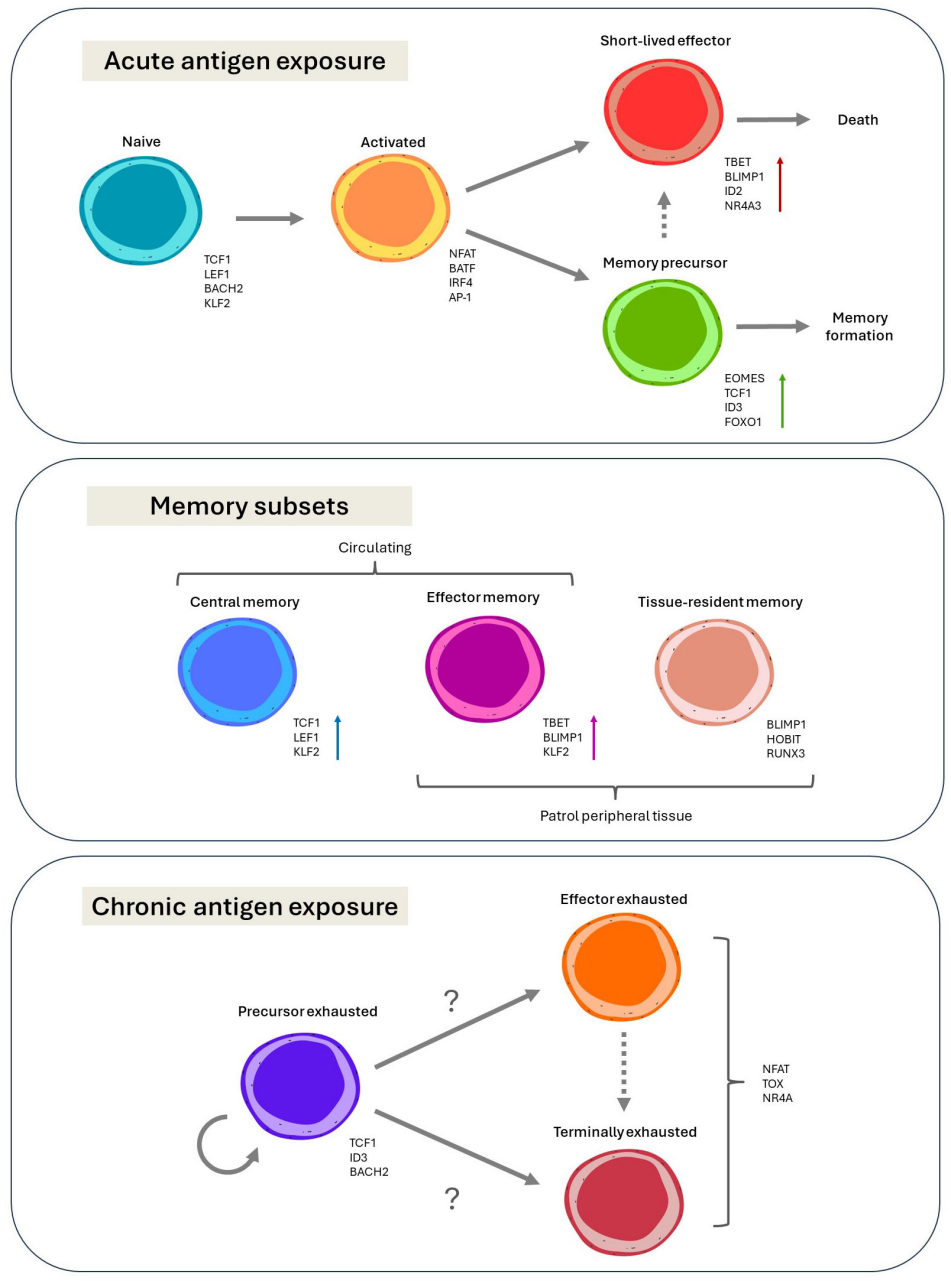
Current paradigms on the diversity and differentiation of CD8^+^ T cells, and the transcription factors associated with the various phenotypic states, with arrows indicating differentiation trajectories.

Transcriptional networks drive strikingly diverse changes in the phenotype and function of a T cell over its lifetime (**Figure 1**). Transcription factors are typically classified as repressors or activators of gene expression, although certain proteins are capable of either activation or repression in different contexts. Modulation of gene expression is mediated by binding at promoters, found near transcriptional start sites, or at enhancers, distal regulatory regions (28). Whilst some transcription factors can operate as single functional units, such as those with a helix-turn-helix (HTH) structure, others must dimerise, such as basic leucine zipper (bZIP) transcription factors e.g. the activating protein 1 (AP-1) family (29, 30). Furthermore, these dimers can bind to additional cofactors to mediate activation or repression at composite element sites. For instance, heterodimers of two AP-1 family members, BATF and JUNB, can bind to interferon regulatory factors (IRF) to modulate transcription at AP-1-IRF composite elements (AICEs) (31). Post-translational modifications play an important role in the regulation of transcription factor activity, with different modifications driving specific outcomes depending on the target protein (32). For example, phosphorylation of nuclear factor of activated T cells (NFAT) sequesters the protein in a resting, cytosolic state, whereas phosphorylation of certain IRFs or signal transducer and activator of transcription proteins (STATs) activates these transcription factors and is required for nuclear translocation (33, 34). Ultimately, the endogenous control of a cell’s transcriptional activity is mediated by the integration of upstream signalling to coordinate appropriate changes in gene expression induced by various stimuli. Accordingly, T cell differentiation is orchestrated by networks of transcription factors that integrate a range of stimuli, such as antigenic stimulation, cellular crosstalk, and environmental cues to allow crucial fate decisions to unfold. Transcriptional networks drive strikingly diverse changes in the phenotype and function of a T cell over its lifetime (**Figure 1**).

In naïve T cells, a maintenance transcriptional program promotes a state of quiescence during its patrol for cognate antigen. This involves the epigenetic and transcriptional silencing of effector-related genes by important transcription factors such as TCF1, BCL2 and LEF1, and maintenance of regular naïve migratory patterns such as suppression of inflammatory chemokine receptors by KLF2 (12, 35–37). Once a T cell receives the three requisite signals of activation, this program is swiftly downregulated, allowing for the transcriptional framework governing effector differentiation and clonal expansion to assume control. TCR-responsive transcription factors such as NFAT and IRF4, and transcription factors induced by co-stimulation, such as c-Jun and NF-κB, integrate to orchestrate the necessary molecular rewiring of the cell (38–41). These transcriptional circuits are further tuned by cytokine support, such as IL-2, IL-7, IL-12, IL-15 and type I IFN signalling (42, 43). With all activating signals delivered to the T cell, these transcriptional networks induce robust proliferation, metabolic rewiring, effector differentiation and the trafficking to sites of pathogenic or malignant insult (44). In contrast, where incomplete signals are delivered, a tolerogenic program is initiated to restrict the T cell response and limit harm to self (41).

In addition to the generation of short-lived effector cells (SLECs), which are purged from the system once the antigenic threat is neutralised, early activated T cells also give rise to a small population of effector cells that are destined to survive contraction and form memory (45, 46). These are known as memory precursor effector cells (MPECs). Their fate is determined by sustained expression of transcription factors driving longevity and stemness, and lower levels of effector-related transcription factors such as IRF4 and BLIMP1 (47, 48). Whilst some of the transcription factors associated with longevity are shared with the naive transcriptional program, such as TCF1, others are uniquely induced after activation, such as EOMES (49–51).

Several transcriptional networks run in parallel during the formation of memory, producing several subsets with unique differentiation states, migratory patterns, and functional capacities to provide comprehensive long-term protection. These include two subsets of circulating populations – central memory T (T_CM_) and effector memory T (T_EM_) cells – and one subset that stably occupies peripheral tissues - tissue resident memory T (T_RM_) cells (52–54). T_CM_ cells adopt similar migratory patterns to that of naive T cells, utilising shared transcription factors such as KLF2 to regulate its course (55). In contrast, low level activity of the effector T cell transcriptional program likely maintains T_EM_ cells in a more terminally differentiated state than T_CM_ cells, and permits trafficking through both blood and peripheral organs (56, 57). Furthermore, a distinct tissue-residency program involving RUNX3, BLIMP1 and HOBIT, governs the T_RM_ compartment, which provides local surveillance at previous sites of antigenic insult (58–60).

In certain disease settings, such as cancer or chronic infection, the epigenetic and transcriptional landscapes of T cells are gradually remodelled to produce a unique phenotypic state known as exhaustion (61). T cell exhaustion is driven by complex mechanisms including chronic antigen exposure, excess of inflammatory signals, and suppressive signals (e.g. cytokines, cell-to-cell signalling) (62). The transcription factor TOX has been identified as an important contributor to the epigenetic ‘scarring’ (63). Over the past few years, our understanding of exhausted T (T_EX_) cells has expanded to appreciate that heterogeneity exists within this T_EX_ population, and that transcription factors likely play a fundamental role in directing these divergent fates. Transcription factors driving quiescence and self-renewal, once again including T cell factor-1 (TCF1), ID3, and BACH2, maintain a progenitor exhausted T (T_PEX_) cell pool (64). These T_PEX_ cells can be a source of sustained anti-tumour activity through the generation of cytotoxic progeny. The definitions surrounding T_EX_ subsets are constantly evolving. However, the field has broadly converged on the existence of at least two distinct fates: (i) effector exhausted cells, thought to provide potent cytotoxic effects; and (ii) terminally differentiated exhausted T cells, which have poor cytotoxicity and are deficient in cytokine production (65–67). Furthermore, a hostile TME alters nutrient and oxygen availability, ultimately affecting the transcriptional program of infiltrating T cells (68, 69). For instance, lower oxygen levels within the TME drives the activity of hypoxia inducible factors, which are thought to allow for cellular adaptations to the challenging environment. In this setting, distinct transcription factors calibrate and fine tune the T cell phenotype in response to chronic exposure to antigen and other factors within the TME.

Transcription factors and their downstream effects thus endow CD8^+^ T cells with an array of phenotypic states. Importantly, certain fates are correlated with better clinical outcomes for patients receiving ACT. For example, stemness – a central feature of naive and central memory CD8^+^ T cells – has been highlighted as a characteristic that correlates with improved CAR-T cell persistence and durable remissions (57, 70). Targeting transcription factors that promote these favourable phenotypes is thus a promising strategy to enhance ACT protocols; this strategy has the potential to attenuate exhaustion-related transcriptional circuits, enhance stemness and/or memory-like potential, and promote tumour accumulation, thus overcoming the various challenges faced when targeting solid tumours. One barrier that is not obviously overcome with transcription factor modulation is tumour heterogeneity. Transcriptional rewiring of T cells into hybrid natural killer (NK)-like phenotypes can overcome MHC-restriction, but does not expand the repertoire of cancer antigens targeted by T cells (71). However, as novel insights into transcription networks involved during successful immunity are unravelled as a consequence of DC-T cell crosstalk, transcription factor targets enhancing important phenomena such as epitope spreading will no doubt assist with overcoming this key obstacle. However, identification of transcription factors that drive effective T cell immunity against cancer remains a significant challenge due to the complexity of its spatiotemporal regulatory patterns during T cell differentiation. Nevertheless, recent years have seen immense advances in molecular and bioinformatics platforms that have been instrumental in garnering mechanistic insight at unprecedented rates.

## 5 Unbiased strategies for prioritising novel transcription factor targets

*In silico* analyses using powerful bioinformatics approaches have accelerated drug discovery beyond the capabilities of traditional ‘reductionist’ (biased) approaches. As such, next generation sequencing technology that captures epigenetic and transcriptomic information (at bulk and single cell resolution) are now at the forefront of cancer immunotherapy research to unlock the heterogeneity of the anti-tumour T cell response. This process typically involves leveraging pre-clinical and clinical models, particularly those with divergent outcomes, and dissecting these models with next generation sequencing to prioritise candidates for downstream experimental validation. Indeed, conventional analysis of transcripts in TILs from human melanoma and non-small cell lung cancer revealed TOX as a critical driver of intratumoral T cell exhaustion which was confirmed by flow cytometry and siRNA knock-out experiments (72). Integrative analysis evaluating ATAC- and RNA-seq data highlighted KLF4 as a novel transcriptional regulator that reinvigorated exhausted CD8^+^ T cells in MC38 murine tumours (67). A similar approach was adopted by Chen et al., reporting the role of NR4A transcription factors in driving CAR-T cell function in humanised animal models for B cell leukaemia (73). New technology involving the simultaneous single cell profiling of chromatin states and RNA in CAR-T cells has also unveiled FOXP1 and KLF2 as reciprocal regulators of stemness and effector function respectively (74). In addition to the more conventional analyses involving these technologies, a suite of *in silico* prediction tools have been specifically developed to garner further mechanistic insight from omics data to identify novel transcriptional regulators. These tools utilise various computational approaches, each with distinct strengths and limitations: 1) Gene regulatory network (GRN) inference methods like ARACNe (75), GENIE3 (76) and WGCNA (77) reveal complex regulatory interactions from transcriptomic data but are computationally intensive and can generate noisy GRNs. 2) Motif enrichment analysis tools, such as HOMER (78), predict TF binding sites through DNA motif analysis, though they may oversimplify predictions and don’t capture secondary or indirect targets. 3) Transcription factor activity scoring approaches, like DoRothEA (79) and VIPER (80) estimate TF activity based on gene expression but is limited in scope due to their reliance on *a priori* information. 4) More advanced tools leverage multi-omic information generated by an array of single cell technologies capturing transcriptomic and epigenetic information. The SCENIC algorithm can predict TF activity from transcriptomic data derived from individual cells (81), but data sparsity inherent to scRNA-seq technology can limit the resolution of transcription factor-target gene predictions. Integrative multi-omic approaches such as MARINa (82) and CellOracle (83) combine gene expression and TF binding data from scRNA-seq and ChIP-seq respectively to generate more robust predictions models. In the context of cancer and T cell biology, the use of tools such as SCENIC and GENIE3, which are computational frameworks that reverse engineer’s gene regulatory networks from transcriptomics data (81), has been instrumental in identifying novel transcriptional networks underlying TIL activity in various human cancer atlas datasets (84, 85), as well as long-term CAR T cell persistence (86). It is important to note that these computational frameworks serve merely as predictive tools, and rigorous laboratory validation is necessary. When combined with *in vitro* high throughput screening tools involving the use of the CRISPR/Cas9 gene editing system, this represents the current state-of-the-art in the field that efficiently and systematically interrogates regulators of T cell fate in cancer. This has been exemplified in numerous studies utilising CRISPR screening that delineate the importance of specific transcription factors programs in tumour-specific CD8^+^ T cell (87, 88) and Treg (89) populations.

## 6 Current tools for the modulation of gene expression

**Figure 2:**
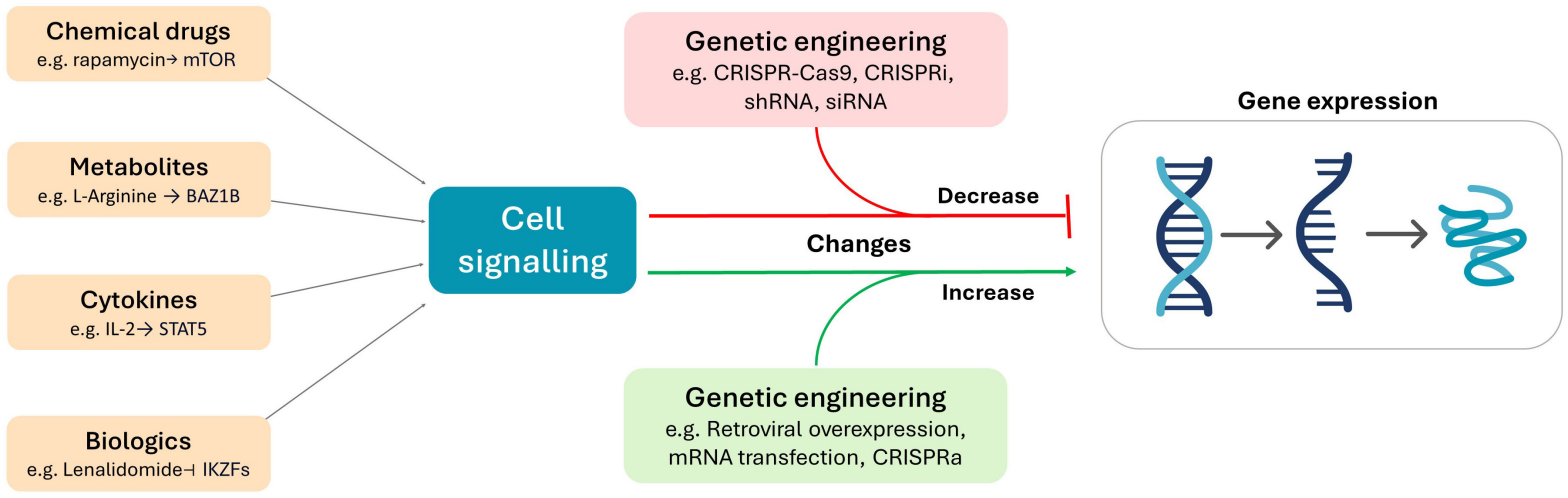
The use of metabolites, chemical drugs, cytokines, and biologics can be used to deliver cellular signals that alter gene expression. In contrast, genetic engineering platforms directly target gene expression by altering or influencing the cell at the genomic (e.g. CRISPR-Cas9, retroviral overexpression) or transcriptomic (e.g. CRISPRi, CRISPRa, shRNA, siRNA, mRNA transfection) level.

A suite of strategies can be used to modulation the expression of transcription factors, such as the use of cytokines (90, 91), chemical drugs (92, 93), immunomodulators (94, 95), and different nutrient levels (96, 97). In contrast to these approaches, which require the identification of biologically active molecules and compounds, genetic engineering platforms are capable of highly targeted manipulation of any known gene (**Figure 2**). The fundamental advantage of genetic engineering strategies is that the transcriptome of the cell becomes largely customisable for the purposes of basic and therapeutic research. The manipulation of transcription factors in CAR T cells can be broadly categorised into approaches aimed at decreasing or enhancing their expression.

Decreasing the expression of transcription factors that give rise to inferior phenotypes can improve CAR-T cell efficacy by diverting cellular products from poor phenotypic subsets and/or promoting effective populations. Several tools exist for the ablation or reduction of gene expression in living systems. The CRISPR-Cas9 system permanently inactivates targeted genes from the cellular genome by introducing loss-of-function mutations (98, 99). CRISPR-Cas9 is favoured for its simplicity, efficiency and accuracy. Novel iterations of CRISPR technology, particularly CRISPR interference (CRISPRi) can also achieve transient repression of target gene transcription (100, 101). The use of small interfering RNAs (siRNA) or short hairpin RNAs (shRNA) can also transiently repress or knockdown (KD) gene expression, although the incorporation of their sequences into viral vectors can establish stable downregulation of target genes (102–105). The Cre/lox system is a sophisticated tool for genetic deletion in mice, that is widely used for its ability to efficiently ablate genes *in vivo*, at designated times, and in designated cellular subsets, by the inducible expression of Cre recombinase under different promoters (106, 107). For example, floxed genes can be deleted in thymocytes at the double positive stage by expression of Cre under the CD4 promoter, in developing or mature T cells by expression of Cre under the proximal or distal Lck promoters, and in activated CD8^+^ T cells by expression of Cre under the Granzyme B promoter (108–110). Whilst the use of CRISPR tools and siRNA or shRNA-based approaches can be feasibly adopted into an ACT protocol, the Cre/lox system can be utilised only in murine models. However, this system has been incredibly valuable for developing fundamental understanding of many genes and continues to be a powerful platform for genetic studies.

In contrast, increasing the expression of transcription factors that drive superior phenotypes can improve CAR T cell efficacy by enriching effective cellular subsets. A frequently used tool is retroviral viral transduction to achieve constitutive overexpression of genes of interest. Benefits of retroviral overexpression include stable and efficient levels of expression (111). Stable expression of genes of interest can also be achieved by HDR-CRISPR approaches to express genes under chosen promoters (112). Although this is a technically challenging approach, it circumvents issues relating to random integration into the genome such as the introduction of dangerous mutations. Ectopic gene expression can additionally be achieved by transfection with mRNA or DNA, although this increased expression is transient in nature (113, 114). Amplification of the wildtype gene can also be achieved through CRISPR activation (CRISPRa) (100, 115). Although there are many novel tools for the introduction or amplification of target genes, retroviral overexpression is most widely used in preclinical studies.

To date, numerous transcription factors have been modulated in CD8^+^ T cells and shown to improve anti-tumour efficacy by conferring the adoptive cellular product with a range of functional or phenotypic advantages (**Table 2**).

**Table 2:** Mechanisms of improved CD8^+^ T cell-driven anti-tumour immunity due to the engineered increased (↑) or decrease (↓) in transcription factor activity.

Family	Protein	Change	Method	Mechanisms of improved anti-tumour response
**NFAT**	NFAT5	**↓**	-/- CD4-Cre (120)	Limits exhaustion
**Tox**	TOX	**↓**	+/- CD4-Cre (63, 124)	Limits exhaustion
	TOX + TOX2	**↓**	shRNA (TOX), genetic deficiency (TOX2) (125)	Limits exhaustion
**NR4A**	NR4A1	**↓**	-/- CD4-Cre (135)	Limits exhaustion
	NR4A3	**↓**	Genetic deficiency (131), CRISPR-Cas9 (141)	Limits exhaustion
	NR4A1, 2, + 3	**↓**	-/- Transduced-Cre (NR4A1, NR4A2), genetic deficiency (NR4A3) (73)	Limits exhaustion
**PRDM**	BLIMP1	**↓**	CRISPR-Cas9 (141, 142), CRISPRi (143)	Limits exhaustion, improves persistence
**JUN**	c-Jun	**↑**	Retroviral overexpression (146, 147)	Limits exhaustion
**TCF/LEF**	TCF1	**↑**	Retroviral overexpression (153)	Limits exhaustion, promotes stemness
**ID**	ID3	**↑**	Retroviral overexpression (155)	Promotes stemness
**MYB**	c-Myb	**↑**	Retroviral overexpression (160)	Promotes stemness
**FOXO**	FOXO1	**↑**	Retroviral and lentiviral overexpression (164, 165)	Promotes stemness and memory phenotypes
**RUNX**	RUNX3	**↑**	Retroviral overexpression (171–173)	Promotes effector function and accumulation
**HIF**	HIF-1α + HIF-2α	**↑**	-/- dLck-Cre or ER-Cre deletion of VHL (177, 178)	Promotes effector function and accumulation
	HIF-2α	**↑**	Retroviral overexpression (180)	Promotes effector function
**ETS**	ETS1	**↓**	CRISPR-Cas9 (87)	Promotes T_EX_ differentiation and function
	FLI1	**↓**	CRISPR-Cas9 (190)	Promotes effector function
**BATF**	BATF3	**↑**	Retroviral overexpression (184)	Promotes memory phenotypes
	BATF	**↑**	Retroviral overexpression (198)	Limits exhaustion
		**↓**	shRNA (195), siRNA (196), CRISPR-Cas9 (197)	Promotes memory phenotypes
**IRF**	IRF4	**↑**	Retroviral overexpression (201)	Enhanced effector function
		**↓**	shRNA (195, 203)	Promotes memory phenotype *(in vitro only)*
**BCL11**	BCL11B	**↓**	CRISPR-Cas9 (208)	Overcomes MHC restriction

CRISPR interference (CRISPRi), knockout of two alleles (-/-), knockout of one allele for haploinsufficiency (+/-), Cre expressed under different conditions, including the CD4 promoter (CD4-Cre), retroviral transduction (Transduced-Cre), tamoxifen-induced (ER-Cre), short hairpin RNA (shRNA).

## 7 Current outcomes in modulating transcription factor expression in CD8^+^ T cells

### 7.1 NFAT

The NFAT transcription factor family is comprised of 5 members, four of which are regulated by the Ca*^2+^*-calcineurin signalling axis (NFATc1-c4), whilst NFAT5 responds to hyperosmotic stress (116–118). Calcium-responsive NFAT isoforms play a fundamental role in initiating early transcriptional responses following TCR signalling (38). NFAT, when combined with other prominent transcriptional regulators such as AP-1 confers a range of effects on T cell phenotype. For instance, AP-1/NFAT cooperation elicits downstream IL-2 and cytokine production (119), whilst NFAT activity alone drives exhaustion-like programs in CD8*^+^* T cells (120). Indeed, deletion of NFAT5 in a CD4 promoter-driven Cre-Lox recombination system improves tumour control by tumour-specific T cells in a mouse model of melanoma (121). Using a same recombination system, Heim et al. demonstrated that the deletion of NFATc1 resulted in ablated memory T cell formation and impaired effector differentiation, highlighting distinct biological activities of NFAT isoforms in driving fate (122).

### 7.2 TOX

The TOX family of transcriptional regulators comprises four members: TOX1 (also referred to as TOX), TOX2, TOX3 and TOX4 (123). In 2019, numerous studies identified and underscored a role for TOX in establishing exhausted T cell populations in viral and cancer studies (63, 124–128). Evidence suggests that the TOX pathway is activated downstream of NFAT during TCR-stimulation, and cooperates with NR4A to establish exhaustion (63, 125). In light of this central role in mediating exhaustion, several studies investigated the effects of knocking out TOX to limit CD8^+^ T cell exhaustion in cancer. Two studies showed that haploinsufficiency of TOX improves *in vivo* anti-tumour efficacy of CD8^+^ T cells in murine models of melanoma and hepatocellular carcinoma (63, 124). Furthermore, KO of TOX2 in addition to shRNA downregulation of TOX in CAR-T cells (*Tox* DKO T cells) further enhanced tumour control (125). Multiomic interrogation of haploinsufficient *Tox^-/+^* and *Tox* DKO T cells typically revealed an effector-like phenotype with reduced features of exhaustion. In contrast, Scott et al. found that T cells ablated of TOX retained features of dysfunction, such as poorer cytokine production, and failed to persist *in vivo*, possibly due to activation-induced cell death (126). This may reflect context-specific differences between studies, as well as complexities of transcriptional circuits governing exhaustion. Furthermore, the unique functional role of TOX2 remains unclear. On one hand, knockout of TOX2 synergises with TOX deficiency to prevent exhaustion, whilst another study suggests that TOX2 may regulate memory formation (125, 129). As such, we are yet to decipher the precise nature of TOX– and TOX2– driven transcriptional networks, however evidence suggests that targeting this axis is indeed a promising strategy to attenuate exhaustion.

### 7.3 NR4A

The NR4A family of transcription factors – NR4A1 (*Nur77*), NR4A2 (*Nurr1*), and NR4A3 (*Nor1*) – are orphan nuclear receptors that operate within several major transcriptional circuits, including induction by NFAT signalling, cooperation with TOX, and negative regulation of Jun and Fos (125, 130). As such, they are involved in multiple aspects of CD8^+^ T cell biology, including the negative selection of autoreactive thymocytes, SLEC formation, establishing peripheral tolerance, and promoting exhaustion (73, 131–134). Thus, NR4As appear to negatively regulate the survival and function of developing and activated CD8^+^ T cell subsets to prevent overactive or inappropriate immune responses. In mice bearing B16-OVA-CD19 melanoma tumours, CD19-targeting CAR-T cells lacking all three NR4As display significantly enhanced control of tumour growth compared to WT CAR-T cells, and were characterised by distinctly less exhausted, more effector-like phenotype capable of increased cytokine production (73). Ablation of individual NR4As, such as NR4A1 and NR4A3 have also been shown to enhance *in vivo* ACT efficacy against lymphoma and melanoma, respectively. In *Nr4a1^-/-^* OT-I cells formed a two to three-fold larger TIL population, reduced expression of exhaustion markers, and greater expression of Bcl-2, IFN-γ, TNF and CD107a (135). *Nr4a3^-/-^* OT-Is are similarly more polyfunctional, but adopt a memory-like phenotype, likely due to the role of NR4A3 in SLEC formation (131). Taken together, these results suggest that targeting the NR4As may generate more effective CAR T cell products.

### 7.4 BLIMP1

B lymphocyte-induced maturation protein-1 (BLIMP1), encoded by the *PRDM1* gene, plays an important role in both B and T cell effector differentiation (136, 137). Upon CD8^+^ T cell activation, BLIMP1 is upregulated in effector cells, particularly SLECs, and controls key effector-related transcriptional events and functions, including the suppression of memory-related genes, induction of cytolytic molecules, and trafficking to tissues (56, 137, 138). In addition, BLIMP1 drives exhaustion in both cancer and viral infection, enforcing a battery of inhibitory genes and repressing stemness, and limiting effector function when expressed at high levels (139, 140). The regulation of Blimp1 expression has thus been investigated as a strategy to enhance CAR-T cell therapy. Two studies have shown that CRIPSR/Cas9 KO of *PRDM1* in CAR-T cells results in improved persistence and overall enhanced tumour control in preclinical models of haematological and solid cancers including leukaemia, melanoma and prostate cancer (141, 142). Both studies showed that knockout of BLIMP1 epigenetically rewired T cells, particularly increasing accessibility at the promoters of stemness and memory-related transcription factors. Interestingly, Jung et al. found that *PRDM1*-ablation also increased accessibility at a set of genes for exhaustion-related transcriptional regulators, suggesting the activation of compensatory exhaustion programs in the absence of BLIMP1 (141). This was circumvented by additional KO of NR4A3, resulting in a further improvements of tumour control. However, both studies also noted that BLIMP1 deficiency impaired cytolytic activity. Strategies to knockdown rather than completely ablate BLIMP1 expression may therefore preserve effector function. Indeed, CRISPRi downregulation of BLIMP1 in human T cells maintains cytotoxicity whilst limiting exhaustion and enriching the central memory subset (143). Overall, targeting the Blimp1 axis may be an effective strategy to combat exhaustion and enhance the persistence of CAR-T cells, although more refined approaches to maximise cytotoxicity whilst suppressing exhaustion may be optimal to best leverage this transcriptional network.

### 7.5 c-Jun

The transcription factor c-Jun is paramount for CD8^+^ T cell activation. c-Jun is induced by costimulation, and cooperates with TCR-induced NFAT to activate the production of IL2, stimulating clonal expansion (144, 145). Thus, c-Jun plays a fundamental role in mediating activation, ensuring the expansion of robust effector populations. Two studies have shown that constitutive expression of c-Jun protects T cells from exhaustion and potently restored polyfunctional cytokine production and improved cellular persistence. This ultimately enhanced *in vivo* control of a range of human cancers, including leukaemia, osteosarcoma, and hepatocellular carcinoma, by CAR-T or TCR-transgenic T cells (146, 147). Evaluation of a panel mutants deficient in transcriptional activation or chromatin binding ability revealed that the functional advantages conferred by c-Jun were at least partly mediated by displacement of AP-1 – IRF complexes from the chromatin, and not direct transcriptional effects (146). Overall, the overexpression of c-Jun reshapes the transcriptional landscape of CD8^+^ T cells towards superior phenotypes, and is a promising target to enhance CAR-T cell performance.

### 7.6 TCF1

TCF1 is a transcription factor that is highly critical in establishing T cell phenotypes that relate to self-renewal, memory, and quiescence (35, 50). TCF1 is highly expressed in naïve T cells, and although silenced over the course of T cell activation it is important for the generation of MPECs in acute infections, and subsequent formation of memory T cell populations (148–150). TCF1 is also a key marker and transcriptional regulator of the T_PEX_ niche, and represses exhaustion-related genes such as Blimp1, IRF4, and NFAT (151, 152). Indeed, ectopic expression or overexpression of TCF1 in tumour-specific T cells has been shown to enhance the formation of the T_PEX_ population, enhance polyfunctionality, reduce expression of inhibitory receptors, and thus improve *in vivo* control of murine melanoma (153). TCF1-overexpressing T cells additionally possess heightened sensitivity to checkpoint blockade. Thus, overexpression of TCF1 can enrich T_PEX_ subsets that give rise to sustained anti-tumour responses.

### 7.7 ID3

Inhibitor of DNA Binding 3 (ID3) regulates properties of stemness and memory in CD8^+^ T cells. Higher levels ID3 expression in early activated CD8^+^ T cells demarcates memory precursors from short-lived effectors, which instead express increased levels of ID2 (138, 154). Furthermore, ID3 expression delineates T_PEX_ populations, and is downregulated in terminally differentiated subsets (151, 155). Thus far, one study has demonstrated that enforcing an ID3-driven transcriptional program can confer resistance to exhaustion in CD8^+^ T cells. CD8^+^ T cells overexpressing ID3 displayed enhanced polyfunctional cytokine secretion, increased cytotoxicity, and increased intratumoral accumulation, ultimately achieving enhanced control of liver tumours in a murine ACT model. Conversely, knockdown of ID3 dampened anti-tumour control and hampered T cell function (155). Therefore, overexpression of ID3 may be a viable strategy to limit exhaustion in CAR-T cells.

### 7.8 c-Myb

Recent studies have revealed that c-Myb, a member of the MYB transcription factor family, plays an important role in regulating properties of stemness and memory in activated CD8^+^ T cells, building on its previously defined role in thymocyte development (156–159). A stem-like CD62L^+^ subset of T_PEX_ cells that mediated effective responses to checkpoint blockade was not only enriched for c-Myb expression, but dependent on c-Myb for its formation (156). Furthermore, memory formation after vaccinia virus infection was significantly impaired when c-Myb was deleted in mature CD8^+^ T cells (160). Evidence suggests that c-Myb potentiates CD8^+^ T cell longevity and survival by driving the expression of anti-apoptotic molecules, driving the expression of TCF1, and repression of ZEB2, a transcription factor that promotes effector differentiation (157, 160). Gautam et al. found that overexpression of c-Myb successfully enforces properties of stemness, which, importantly, generates more effective anti-tumour responses. c-Myb overexpression improved the metabolic fitness and polyfunctionality of CD8^+^ T cells, generated greater numbers of stem-like populations, and preserved CD62L expression after repetitive stimulation, a process that typically drives terminal effector differentiation. Adoptively transferred c-Myb overexpressing CD8^+^ T cells provided curative anti-tumour immunity in mice bearing melanoma tumours. Furthermore, c-Myb overexpressing cells formed effective memory populations that protected hosts against the development of tumours upon a secondary melanoma challenge (160). As such, ectopic expression of c-Myb is an attractive approach to enhance stem-like phenotypes that promote persistence and superior anti-tumour responses in CD8^+^ T cell ACT products.

### 7.9 FOXO1

FOXO1 is an important transcriptional regulator of stemness in both naive and antigen-experienced CD8^+^ T cells and is crucial for the establishment of memory and long-term survival (161–163). Two recent studies found that direct overexpression of FOXO1 in CAR-T cells improves anti-tumour efficacy (164, 165). Phenotypically, human CAR-T cells overexpressing FOXO1 were enriched for markers of memory, and decreased levels of exhaustion. FOXO1-overexpressing CAR-T cells additionally demonstrated increased expansion, and greater polyfunctionality. Metabolically, FOXO1-overexpressing CAR-T cells demonstrated oxidative phosphorylation than control cells, indicating superior cellular fitness. Interestingly, both studies found that using a constitutively active version of FOXO1 that was insensitive to nuclear export resulted in blunted production of cytokines by the CAR-T cells. In addition, use of an EF1α promoter which drove higher expression levels of wildtype FOXO1 resulted in a stronger memory-like phenotype as compared to expression driven by other promoters (165). These findings elegantly demonstrate that balanced and fine-tuned expression of FOXO1 maximized the functional and phenotypic advantages conferred to CAR-T cells. Finally, when evaluated in an *in vivo* setting, FOXO1 overexpression improved CAR-T cell anti-tumour control in a range of preclinical solid tumour models, including murine breast carcinoma and colon adenocarcinoma, and human ovarian cancer and osteosarcoma. Importantly, these studies provided direct comparisons between FOXO1 and TCF1-overexpressing CAR-T cells, demonstrating FOXO1 reduced exhaustion, improved expansion, and enhanced *in vivo* outcomes in a model of leukaemia (164, 165)vghy. Numerous studies have now identified that overexpression of various master regulators of memory and stemness can indeed improve the therapeutic efficacy of CAR-T cell therapy in several preclinical models, and experiments to identify the most effective of these transcription factors are of utmost importance.

### 7.10 RUNX3

RUNX3 governs multiple aspects of CD8^+^ T cell fate decisions, from commitment to the CD8^+^ lineage during thymopoiesis, to governing transcriptional networks post-activation (166–168). RUNX33 is critical for formation of functional memory T cells, underpinning widespread chromatin remodelling upon TCR stimulation including increased accessibility at effector-related genes such as *Irf4*, *Prdm1*, *Id2*, *Eomes*, and *Il2ra* (167). Recently, RUNX3 has additionally been highlighted for its key role in establishing T_RM_ subsets in CD8^+^ T cells, driving tissue residency in a range of tissues (58, 169, 170). Exploiting RUNX3-driven transcriptional networks is thus an attractive strategy to enhance CAR-T cell therapy, as accumulation within the tumour bed is imperative for CD8^+^ T cell-mediated tumour control. In a model using murine P14 T cells targeting GP_33_-expressing B16 melanoma, overexpression of RUNX3 drove greater abundance of TILs, granzyme B expression, and tissue-residency gene modules to ultimately delay tumour growth and prolong survival (171, 172). RUNX3-overexpression synergises with inhibition of protein kinase B (Akt) to promote additional T_CM_ differentiation in CAR-T cells, which produces robust and improved anti-tumour responses in pancreatic ductal adenocarcinoma patient-derived xenograft tumour models (173). However, these results conflict with a study using a different human CAR-T cell model, which found that RUNX3-overexpression in anti-mesothelin CAR-T cells neither promoted a tissue-residency phenotype, nor improved control of mesothelioma *in vivo* (174). Thus, RUNX3 is a promising target that may engineer CAR-T cells with favourable characteristics such as tissue residency features and improved persistence to achieve superior therapeutic outcomes, although evidence is unclear as to whether these outcomes are consistent across multiple models and systems.

### 7.11 HIF-1α and HIF-2α

Hypoxia inducible factors 1 and 2 alpha (HIF-1α and HIF2α) are dimeric transcription factors that mediate homeostatic responses to low oxygen levels, such as those found within the tumour microenvironment (175, 176). In addition to governing homeostatic transcriptional circuitry, HIFs support CD8^+^ effector cell differentiation and tissue resident fates (177–179). Two studies demonstrated that deletion of the von Hippel-Lindau tumour suppressor gene (VHL), a negative regulator of HIF, to promote HIF activity improves the anti-tumour immunity of CD8^+^ T cells against primary and metastatic melanoma, and colorectal cancer in murine models. Unrestrained HIF activity enhanced effector functions, particularly cytotoxicity and cytokine production, along with increased transcription of inhibitory molecules and increased protein expression of costimulatory markers (177, 178). VHL deletion could also generate T_RM_-like TILs with superior accumulation and survival within tumours. Therefore, genetic engineering platforms to reduce or ablate VHL in CAR-T cells, such the use of CRISPR knockout or interference tools, may improve anti-tumour efficacy by enforcing HIF-driven transcriptional circuits. Veliça et al. showed that ectopic overexpression of HIF-2α, particularly a factor inhibiting HIF (FIH)-insensitive mutant, was shown to more effectively enhance their anti-tumour efficacy (180). These CD8^+^ T cells with enhanced cytotoxicity, increased expression of costimulatory molecules, and greatest *in vivo* efficacy against melanoma and leukaemia. This suggests unknown complexities in this transcriptional circuit, including the differences in HIF-1α vs HIF-2α driven-networks, and the nuances of different regulatory systems. Overall, the same cellular machinery used to adapt to hypoxia can drive improved effector phenotypes that benefit CAR-T cells for tumour elimination.

### 7.12 BATF3

The basic leucine zipper transcriptional factor ATF-like (BATF) family of transcription factors includes BATF1, BATF2 and BATF3 (181). Unlike BATF1 (discussed below), CD8^+^ T cell-intrinsic BATF3 is non-essential for mounting the primary response to infection, however it is important for the establishment of memory CD8^+^ T cell populations to ensure effective recall responses, provides protection against apoptosis, and maintains cellular fitness (182, 183). Overexpression of BATF3 promotes CD8^+^ T cell survival and enforces a battery of genes associated with the establishment and maintenance of memory phenotypes as well as CD8^+^ T cell function (182). In a pipeline for identifying transcription factors regulating memory phenotypes in human CD8^+^ T cells, BATF3 was identified as a positive regulator of memory-like phenotypes and cellular fitness, amongst other more characterised proteins such as MYB and FOXO1. Overexpression of BATF3 suppressed CAR-T cell exhaustion and enhanced *in vivo* tumour control in an orthotopic breast cancer model (184). However, a recent report showed that unrestrained BATF3 due to loss of TET2 drove clonal expansion of CAR-T cells (185). Given concerns about the safety of engineered cellular products, particularly with respect to the development of secondary cancers derived from the infused cells, it is important to approach potent drivers of proliferation with caution. To overcome such issues, transient expression systems, as discussed later in this review, may be useful tools to create safer, more controlled CAR-T cells.

### 7.13 ETS1

ETS1 is an important enforcer of quiescence, driving other stem-like transcription factors such as TCF1 and BCL6, whilst repressing effector molecules and regulators such as CD25 and BLIMP1 (186, 187). Although transiently downregulated during activation, ETS1 deficiency compromises the survival and proliferation of murine T cells during activation (188). In activated T cells, ETS1 is important for the maintenance of IL-7 receptor, which regulates survival and homeostasis in memory CD8^+^ T cells (189). Recently, Zhou et al. utilised a single-cell CRISPR screening platform to unravel transcriptional regulators of diverse CD8^+^ TIL fates, particularly those that governed transitions between different T_PEX_ and T_EX_ states. ETS1 was identified as a key repressor of differentiation from T_PEX_ into T_EX_, and CRISPR-Cas9 KO of ETS1 in activated CD8^+^ T cells improve anti-tumour efficacy in a suite of murine melanoma models, alongside increased cytokine production and cytotoxicity (87). Whilst many strategies revolve around enforcing memory or stem-like populations, this approach highlights the importance and advantages of permitting transition from quiescent phenotypes into differentiated subsets with effector capacities.

### 7.14 FLI1

FLI1 is another member of the ETS transcription factor family, however with a largely unexplored role in CD8^+^ T cell biology. In a CRISPR screening platform, FLI1 was identified as a negative regulator of effector subsets. Consequently, KO of FLI1 generated robust effector CD8^+^ T cell populations that displayed enhanced control of tumours and various pathogens, whilst limiting T_PEX_ numbers. Furthermore, FLI1 epigenetically restricted accessibility at ETS: RUNX binding sites, which were then exposed upon FLI1 ablation. This epigenetic remodelling due to KO of FLI1 synergised with overexpression of RUNX3 to further enforce effector subsets (190). Thus, CRISPR screens have successfully identified two members of the ETS transcription factor family as actionable molecular targets to promote effective CD8^+^ T cell responses (87, 190). Manipulation of these transcriptional networks may enhance the performance of CAR T cells via the bolstering of effector activity.

### 7.15 BATF

BATF1, typically referred to as BATF, is critical for the commitment of CD8^+^ T cells to the effector lineage as well as the proliferative burst following activation (191–193). The activity of BATF can involve complexing with partner transcription factors, most notably IRF4, as well as other AP-1 family members such as c-Jun, to promote the effector transcriptional program (193). In addition to its role in the effector T cell response, BATF expression has been correlated with CD8^+^ T cell exhaustion, however a causal relationship has not been determined (194, 195). Interestingly, studies have shown both up- and down- regulation of BATF in CD8^+^ T cells can improve anti-tumour immunity. Silencing or knockdown of BATF promoted memory-like phenotypes capable of superior anti-tumour immunity in a range of ACT models, including patient-derived pancreatic carcinomas (195–197). On the contrary, a recent paper by Seo et al. demonstrated that constitutive overexpression of BATF attenuated T cell exhaustion and drove superior anti-tumour control by OT.Is and CAR-T cells in preclinical mouse melanoma models. However, instead of promoting stem-like features, overexpression of BATF reinforced effector-like phenotypes (198). Notably, BATF has been shown to drive the differentiation of effector-like CX_3_CR1^+^ cells in chronic LCMV infection (199). This suggests a potential mechanism by which BATF overexpression in anti-tumour CD8^+^ T cells generates robust responses. Thus, because BATF is both a driver of effector differentiation and a suppressor of memory, its transcriptional circuits may be activated to generate more effector-like CD8^+^ T cells, or repressed to generate more memory-like CD8^+^ T cells, ultimately improving anti-tumour efficacy.

### 7.16 IRF4

IRF4 is a TCR-responsive transcription factor that plays a dual role in CD8^+^ T cell effector and exhaustion programs (39, 200). IRF4 is necessary for the differentiation of fully functional effector CD8^+^ T cells, maximal proliferation, cytokine production, cytotoxicity, and thus critical for effective responses to bacterial, viral, and malignant threats (39, 47, 193). The role of IRF4 in CD8^+^ T cell exhaustion is nuanced, with several studies point towards an exhaustion-inducing function of IRF4, whilst others argue that it is instead crucial for sustained cytotoxic activities (195, 200–202). In an *in vitro* model of artificially induced CAR-T cell exhaustion, IRF4 was upregulated in terminally exhausted CD8^+^ T cells, and shRNA knockdown of IRF4 reduced inhibitory receptor expression, and enriched the T_CM_-like population (195, 203). In contrast, another study showed that depletion of IRF4 using the DTR system compromised tumour control of by endogenous CD8^+^ T cells and impaired the efficacy of adoptively transferred T cells (202). Thus, it remains unclear how to optimally leverage the activity of IRF4 in anti-tumour T cells. Interrogation of its downstream targets may reveal distinct circuits for driving effector responses over exhaustion. As novel genetic engineering platforms emerge alongside increased understanding of these circuits, it may be possible to more finely tune this axis to maximise anti-tumour responses.

### 7.17 BCL11B

BCL11B is a transcription factor that protects the T cell identity during development, particularly safeguarding from the NK lineage (204, 205). Interestingly, transcriptional networks that orchestrate immune cell lineages can be manipulate to induce transdifferentiation between different immune cell types (206). Indeed, downregulation or loss of BCL11B in T cells generates induced-T cells with NK-like features (ITNKs) that acquire an NK-like genetic signature and express functional NK receptors. This has been demonstrated in several models and at various stages of T cell differentiation, including engineered deletion using Cre/lox systems at different stages of thymopoiesis (71), and an expanded BCL11B^low^ T cell subset in human cytomegalovirus-seropositive individuals (207). Importantly, ITNKs generated by CRISPR-Cas9 deletion of BCL11B in peripheral blood-derived human T cells demonstrate robust anti-tumour activities against leukemic cell lines *in vitro* and *in vivo* (208). Deletion of BCL11B additionally enables killing of MHC-low or MHC-negative cancer cells. Furthermore, *in vivo* anti-tumour activity against leukemic and hepatocellular carcinoma of ITNKs was enhanced when equipped with a CAR targeting CD19 or glypican-3, respectively (208). Thus, reprogramming of T cells with a hybrid phenotype that leverages both innate and adaptive lymphocyte qualities is a novel strategy to enhance the anti-tumour activity of CAR-T cells.

## 8 Advancements into clinical trials

Preclinical screening pipelines and novel technologies have successfully identified a number of candidate transcription factors that can be modulated to confer CAR-T cells with improved efficacy against solid tumours. To date, two of these discussed targets have advanced into preliminary phase I clinical trials. RUNX3-overexpressing CAR-T cells have been assessed for their safety profile in the treatment of metastatic hepatocellular carcinomas, demonstrating manageable toxicities thus far (209). The safety and tolerability of ITNKs, generated by BCL11B deletion, are being investigated for the treatment of a range of MHC-low or MHC-negative advanced solid tumours, and preliminary results from nine patients have been reported (208, 210). Thus far, clinical benefit (either disease stabilisation or partial remission in one case) has been observed in two thirds of treated patients, with no toxicities experienced (208). As such, transcription factor-modulated T cell products are successfully progressing into human trials, demonstrating the feasibility of generating such products. Comparisons with non-transcription factor-modulated products will be highly interesting to define the therapeutic benefit provided by rewiring these T cell phenotypes.

## 9 Future considerations

### 9.1 Targeting transcription factor combinations to improve ACT

Transcription factors in cells operate in a highly cooperative and interconnected fashion, with different binding partners or co-factors influencing the biological effects exerted. An effective strategy of rewiring anti-tumour immunity may therefore require modulating multiple transcription factors rather than a single target in isolation. Targeting combinations of transcription factors may additionally address redundancies in the biological circuitry of the cell. For example, the BLIMP1/NR4A3 dual KO (141), TOX/TOX2 dual KO (125), or NR4A triple KO T cells (73) all outperformed single KO counterparts in their respective studies. Another potential approach may be the disruption of specific transcriptional complexes. Mognol et al. used a FRET-based high-throughput screen to identify a compound that effectively disrupted NFAT: AP-1 complexes at target DNA binding sites (211). It would be interesting if this compound could redirect T cells from an activated phenotype towards tolerance or dysfunction. This could be therapeutically relevant for immunopathology caused by excessive T cell-mediated activity, such as in autoimmune diseases like multiple sclerosis or type 1 diabetes (212, 213). Alternatively, to enhance CD8^+^ T cell responses for elimination of malignant cells, compounds that, for example, stabilised NFAT: AP1 interactions, or targeted other important transcriptional complexes, could be a promising approach for fine-tuning the molecular circuitry in T cells.

### 9.2 Transient TF expression

Genetic engineering platforms continue to advance at a rapid pace, providing cellular therapies with increasingly sophisticated tools. New methods may be capable of achieving more tailored transcriptional programming to better maximise CD8^+^ T cell responses. As these methods emerge, we may find that more simplistic means of modulating gene expression such as overexpression or CRISPR KOs lack the ability to capitalise on complex biological circuits that dynamically regulate the transcriptome. For example, although promoting memory-like phenotypes is a promising approach of improving the persistence and overall functionality of T cells, transcription factors that establish memory phenotypes can work to dampen effector functions (214, 215). Could constitutive activation of memory transcriptional programs oppose full recovery of CD8^+^ T cell effector function upon antigenic stimulation? Alternatively, attenuating T cell exhaustion often relies on downregulation of transcription factors such as BATF, NFAT and BLIMP1 which typically have a dual role in promoting effective effector responses and/or activation (137, 141, 191, 195). Could constant repression or total ablation of these transcription factors be partially limiting effector responses? Furthermore, complete ablation or constitutive overexpression of transcription factors with tumour-suppressor functions (e.g. BLIMP1 (216)) or oncogenic potential (e.g. BATF3 (185, 217)) may be dangerous in modifying a cellular product. A more refined approach to enhancing T cell anti-tumour immunity may instead be temporal, context-dependent modulation of transcription factors as and when they are needed.

Druggable systems exist, such as the Tet-On/Off system, which allow for manual control of gene expression (218). Indeed, one study demonstrated that using 4-hydroxytamoxifen, zinc finger transcriptional activation of an anti-CD20 CAR could achieve titratable CAR expression *in vitro* and *in vivo* (219). However, to extrapolate druggable approaches such as this to expression of transcription factors may be unfeasible, as it may prove to be time consuming and challenging to determine dosing strategies.

Several novel strategies are emerging to achieve inducible gene expression. One such strategy is the design of ‘logic-gated’ CARs, which upregulate gene programs in response to specific environmental stimuli (220–222). For instance, synthetic Notch CAR-T cells have been engineered to upregulate CAR expression upon recognition of tumour antigens by a synthetic Notch receptor. This approach circumvented issues associated with tonic CAR signalling, generating more persistent cellular products that exhibited enhanced anti-tumour control in preclinical solid tumour models (222). Synthetic Notch receptors are extremely versatile tools. The intracellular domain of such receptors have been engineered to contain transcription factors directed towards reporter genes to provide functional readouts (223). Another strategy is knocking-in or inserting a gene of interest into the locus of an endogenous gene, such that the expression of the inserted gene is under the control of the promoter of the endogenous gene. This was successfully performed by Kim et al. The IL-12 gene was inserted into the PD-1 locus in T cells to achieve IL-12 expression upon T cell activation and target-cell recognition whilst simultaneously disrupting the wildtype PD-1 receptor, overall enhancing the effector function and anti-tumour responses of T cells (224). Degron technologies that exploit protein degradation pathways to rapidly target and ablate proteins can also be used. Jan et al. tagged a CAR construct with the degron of the IKZF3 protein, which can be targeted using lenalidomide, a pharmacological mediator of IKZF3 and several other proteins degradation, to induce rapid degradation of the CAR (225). Another form of degron technology called the bioPROTAC, which binds and tags a degron to a target protein, has been combined with synthetic Notch receptors in a CAR construct, to generate an inducible system modulating endogenous levels of a protein of interest in tandem with CAR expression (226). Repurposing inducible systems such as synthetic Notch receptors, degron technologies, and site-specific knock-ins, to modulate the expression of transcription factors that drive superior anti-tumour CD8^+^ T cell responses may be promising approaches for achieving dynamic transcriptional modulation.

### 9.3 Tissue specificity

Another interesting prospect for the field is the possibility of engineering tissue-specific CAR-T cells. As highlighted previously, transcription factors such as RUNX3 can be targeted to drive accumulation of T cells in tumour sites (59). Transcriptional programs driving T_RM_ cells have been shown to overlap with programs in TILs, which is coherent with these populations sharing a common propensity for tissue accumulation and retention, as opposed to recirculation. Interestingly, it has been shown that immune cells in different tissues have specific transcriptional and metabolic programs that promote their residency within these distinct niches. Whether this is due to specific trafficking processes, survival capabilities within their unique metabolic milieu, or other phenomena, is unknown. These unique, tissue tropic transcriptional modules have been investigated in a range of immune cells including macrophages (227) and ILC2s (228). Recently, a study identified a transcriptional network that governed tissue-specific residency of CD8^+^ T cells in the small intestine (229). Targeting tissue tropism may be one strategy to enhance ACT towards tissue specific tumours. Genetic engineering strategies could co-opt tissue-specific transcriptional networks in immune cells to generate cells that specifically hone to a cancer’s tissue of origin. For instance, could engineering of liver-specific tissue residency transcription factors create liver-tropic CAR-T cells that more effectively control hepatocellular carcinomas? Could this strategy be used to target metastatic disease that is almost exclusively observed in one organ, such as uveal melanoma metastases present in the liver? As the field unravels the complexities of residency within the diverse and distinct niches of the body, new molecular targets to create tailored CAR-T cell therapies may emerge.

### 9.4 Looking beyond CAR-T cell therapy and cancer

Finally, although CAR-T cell therapy for cancer draws immense attention in preclinical research and in the clinic, other forms of cell therapies exist for the treatment of cancers and other diseases. The rewiring of transcriptional circuitry may be a powerful tool across these various platforms. TIL therapy is a prime example of another T cell-based therapy that may be enhanced by reshaping the transcriptional landscape. TIL therapy is used for the treatment of relapsed or refractory metastatic melanomas, outperformed anti-CTLA treatment in a Phase 3 clinical trial, and has recently been approved for the clinic by the FDA (230–232). TILs from patient data sets and preclinical murine models provide valuable tools for unravelling tumour-specific T cell exhaustion (233, 234). As such, the targets identified from these data sets may be directly applicable to TIL therapy, counteracting or reinforcing highly relevant biological circuits in these cells. For example, CRISPR/Cas9 KO of *PRDM1* in human TILs can restore polyfunctional cytokine production and expression of memory surface markers CD62L and CCR7 (142). In addition, other immune cells such as NK cells and macrophages are also emerging as promising alternatives to T cell therapies, and transcriptional rewiring may be an attractive approach to further enhance their respective anti-tumour capabilities (235–237). Recently, ID3 expression was found to program effective anti-tumour macrophages (238). Thus, transcriptional modulation to enhance cell therapies may be a versatile strategy that extends beyond cancer, and beyond T cells. CAR-T cell therapies are also under development for pathologies beyond cancer (239–241). Recent clinical trials have demonstrated therapeutic efficacy using CAR-T cells for HIV and various autoimmune diseases (242, 243). Importantly, many major discoveries regarding T cell fate decisions and their governing transcriptional networks are owed to pioneering fundamental research in the anti-viral immunity field. Anti-viral cell therapies may benefit from exploiting these transcriptional circuits that have already been characterised in a viral context, to provide superior future outcomes for patients with chronic infections such as HIV.

## 10 Conclusions

Major advances in powerful sequencing technologies have been instrumental in unravelling the intricate transcriptional networks that govern the phenotype and function of CD8^+^ T cells. This accelerated discovery of genetic targets is met with concurrent developments in genetic engineering platforms that allow us to leverage these molecular drivers with increasing efficiency and accuracy. Preclinical screening pipelines have demonstrated that targeting the expression of transcription factors in CAR-T cell products is an effective approach to overcome existing barriers in the treatment of solid tumours, with emerging clinical data demonstrating that this strategy is feasible in the clinic. Thus, emerging strategies to enhance CAR-T cell therapy hold promise for improving clinical outcomes to patients with cancer.

## References

[1] Waldman AD Fritz JM Lenardo MJ. A guide to cancer immunotherapy: from T cell basic science to clinical practice. Nat Rev Immunol. (2020) 20:651–68. doi: 10.1038/s41577-020-0306-5 PMC7238960 32433532

[2] Tan S Day D Nicholls SJ Segelov E. Immune checkpoint inhibitor therapy in oncology. JACC CardioOncol. (2022) 4:579–97. doi: 10.1016/j.jaccao.2022.09.004 PMC9830229 36636451

[3] Monberg TJ Borch TH Svane IM Donia M. TIL therapy: facts and hopes. Clin Cancer Res. (2023) 29:3275–83. doi: 10.1158/1078-0432.CCR-22-2428 37058256

[4] Sterner RC Sterner RM. CAR-T cell therapy: current limitations and potential strategies. Blood Cancer J. (2021) 11:1–11. doi: 10.1038/s41408-021-00459-7 33824268 PMC8024391

[5] D’Angelo SP Araujo DM Abdul Razak AR Agulnik M Attia S Blay J-Y. Afamitresgene autoleucel for advanced synovial sarcoma and myxoid round cell liposarcoma (SPEARHEAD-1): an international, open-label, phase 2 trial. Lancet. (2024) 403:1460–71. doi: 10.1016/S0140-6736(24)00319-2 PMC11419333 38554725

[6] Martin MD Badovinac VP. Defining memory CD8 T cell. Front Immunol. (2018) 9:2692. doi: 10.3389/fimmu.2018.02692 30515169 PMC6255921

[7] Gebhardt T Park SL Parish IA. Stem-like exhausted and memory CD8+ T cells in cancer. Nat Rev Cancer. (2023) 23:780–98. doi: 10.1038/s41568-023-00615-0 37821656

[8] Takahashi K Yamanaka S. Induction of pluripotent stem cells from mouse embryonic and adult fibroblast cultures by defined factors. Cell. (2006) 126:663–76. doi: 10.1016/j.cell.2006.07.024 16904174

[9] Takahashi K Tanabe K Ohnuki M Narita M Ichisaka T Tomoda K. Induction of pluripotent stem cells from adult human fibroblasts by defined factors. Cell. (2007) 131:861–72. doi: 10.1016/j.cell.2007.11.019 18035408

[10] Taniuchi I Ellmeier W. Chapter 3 - Transcriptional and Epigenetic Regulation of CD4/CD8 Lineage Choice. In: Alt FW Austen KF Honj T Melchers F Uhr JW Unanue ER, editors. Advances in Immunology. Academic Press (2011). p. 71–110. doi: 10.1016/B978-0-12-387663-8.00003-X 21762816

[11] Germain RN. T-cell development and the CD4–CD8 lineage decision. Nat Rev Immunol. (2002) 2:309–22. doi: 10.1038/nri798 12033737

[12] Bennett TJ Udupa VAV Turner SJ. Running to stand still: naive CD8+ T cells actively maintain a program of quiescence. Int J Mol Sci. (2020) 21:9773. doi: 10.3390/ijms21249773 33371448 PMC7767439

[13] Rohaan MW Wilgenhof S Haanen JBAG. Adoptive cellular therapies: the current landscape. Virchows Arch. (2019) 474:449–61. doi: 10.1007/s00428-018-2484-0 PMC6447513 30470934

[14] Shah NN Fry TJ. Mechanisms of resistance to CAR T cell therapy. Nat Rev Clin Oncol. (2019) 16:372. doi: 10.1038/s41571-019-0184-6 30837712 PMC8214555

[15] Guzman G Reed MR Bielamowicz K Koss B Rodriguez A. CAR-T therapies in solid tumors: opportunities and challenges. Curr Oncol Rep. (2023) 25:479–89. doi: 10.1007/s11912-023-01380-x PMC10110629 36853475

[16] Annesley C. Phase 1 study of B7-H3, EGFR806, HER2, and IL13-zetakine (Quad) CAR T cell locoregional immunotherapy for pediatric diffuse intrinsic pontine glioma, diffuse midline glioma, and recurrent or refractory central nervous system tumors. clinicaltrials.gov, National Library of Medicine (2024). Available at: https://clinicaltrials.gov/study/NCT05768880.

[17] Lai J Mardiana S House IG Sek K Henderson MA Giuffrida L. Adoptive cellular therapy with T cells expressing the dendritic cell growth factor Flt3L drives epitope spreading and antitumor immunity. Nat Immunol. (2020) 21:914–26. doi: 10.1038/s41590-020-0676-7 32424363

[18] Etxeberria I Bolaños E Quetglas JI Gros A Villanueva A Palomero J. Intratumor adoptive transfer of IL-12 mRNA transiently engineered antitumor CD8+ T cells. Cancer Cell. (2019) 36:613–629.e7. doi: 10.1016/j.ccell.2019.10.006 31761658

[19] Wang Y Wang J Yang X Yang J Lu P Zhao L. Chemokine receptor CCR2b enhanced anti-tumor function of chimeric antigen receptor T cells targeting mesothelin in a non-small-cell lung carcinoma model. Front Immunol. (2021) 12:628906. doi: 10.3389/fimmu.2021.628906 33777013 PMC7992009

[20] Sagnella SM White AL Yeo D Saxena P van Zandwijk N Rasko JEJ. Locoregional delivery of CAR-T cells in the clinic. Pharmacol Res. (2022) 182:106329. doi: 10.1016/j.phrs.2022.106329 35772645

[21] Dong X Ren J Amoozgar Z Lee S Datta M Roberge S. Anti-VEGF therapy improves EGFR-vIII-CAR-T cell delivery and efficacy in syngeneic glioblastoma models in mice. J Immunother Cancer. (2023) 11:e005583. doi: 10.1136/jitc-2022-005583 36898734 PMC10008211

[22] Liu X Zhang Y Li K Liu Y Xu J Ma J. A novel dominant-negative PD-1 armored anti-CD19 CAR T cell is safe and effective against refractory/relapsed B cell lymphoma. Transl Oncol. (2021) 14:101085. doi: 10.1016/j.tranon.2021.101085 33813229 PMC8050776

[23] McGowan E Lin Q Ma G Yin H Chen S Lin Y. PD-1 disrupted CAR-T cells in the treatment of solid tumors: Promises and challenges. BioMed Pharmacother. (2020) 121:109625. doi: 10.1016/j.biopha.2019.109625 31733578

[24] Alizadeh D Wong RA Yang X Wang D Pecoraro JR Kuo C-F. IL15 enhances CAR-T cell antitumor activity by reducing mTORC1 activity and preserving their stem cell memory phenotype. Cancer Immunol Res. (2019) 7:759–72. doi: 10.1158/2326-6066.CIR-18-0466 PMC6687561 30890531

[25] Watanabe N Mo F McKenna MK. Impact of manufacturing procedures on CAR T cell functionality. Front Immunol. (2022) 13:876339. doi: 10.3389/fimmu.2022.876339 35493513 PMC9043864

[26] Forsberg EMV Lindberg MF Jespersen H Alsén S Bagge RO Donia M. HER2 CAR-T cells eradicate uveal melanoma and T-cell therapy–resistant human melanoma in IL2 transgenic NOD/SCID IL2 receptor knockout mice. Cancer Res. (2019) 79:899–904. doi: 10.1158/0008-5472.CAN-18-3158 30622115

[27] Forsberg EMV Riise R Saellström S Karlsson J Alsén S Bucher V. Treatment with anti-HER2 chimeric antigen receptor tumor-infiltrating lymphocytes (CAR-TILs) is safe and associated with antitumor efficacy in mice and companion dogs. Cancers (Basel). (2023) 15:648. doi: 10.3390/cancers15030648 36765608 PMC9913266

[28] Lambert SA Jolma A Campitelli LF Das PK Yin Y Albu M. The human transcription factors. Cell. (2018) 172:650–65. doi: 10.1016/j.cell.2018.01.029 PMC12908702 29425488

[29] Aravind L Anantharaman V Balaji S Babu MM Iyer LM. The many faces of the helix-turn-helix domain: Transcription regulation and beyond☆. FEMS Microbiol Rev. (2005) 29:231–62. doi: 10.1016/j.fmrre.2004.12.008 15808743

[30] Fujii Y Shimizu T Toda T Yanagida M Hakoshima T. Structural basis for the diversity of DNA recognition by bZIP transcription factors. Nat Struct Mol Biol. (2000) 7:889–93. doi: 10.1038/82822 11017199

[31] Glasmacher E Agrawal S Chang AB Murphy TL Zeng W Vander Lugt B. A genomic regulatory element that directs assembly and function of immune-specific AP-1–IRF complexes. Science. (2012) 338:975–80. doi: 10.1126/science.1228309 PMC5789805 22983707

[32] Filtz TM Vogel WK Leid M. Regulation of transcription factor activity by interconnected, post-translational modifications. Trends Pharmacol Sci. (2014) 35:76–85. doi: 10.1016/j.tips.2013.11.005 24388790 PMC3954851

[33] Honda K Takaoka A Taniguchi T. Type I inteferon gene induction by the interferon regulatory factor family of transcription factors. Immunity. (2006) 25:349–60. doi: 10.1016/j.immuni.2006.08.009 16979567

[34] Sharma S Findlay GM Bandukwala HS Oberdoerffer S Baust B Li Z. Dephosphorylation of the nuclear factor of activated T cells (NFAT) transcription factor is regulated by an RNA-protein scaffold complex. Proc Natl Acad Sci U.S.A. (2011) 108:11381–6. doi: 10.1073/pnas.1019711108 PMC3136327 21709260

[35] Shan Q Li X Chen X Zeng Z Zhu S Gai K. Tcf1 and Lef1 provide constant supervision to mature CD8+ T cell identity and function by organizing genomic architecture. Nat Commun. (2021) 12:5863. doi: 10.1038/s41467-021-26159-1 34615872 PMC8494933

[36] Wojciechowski S Tripathi P Bourdeau T Acero L Grimes HL Katz JD. Bim/Bcl-2 balance is critical for maintaining naive and memory T cell homeostasis. J Exp Med. (2007) 204:1665–75. doi: 10.1084/jem.20070618 PMC2118628 17591857

[37] Willinger T Freeman T Herbert M Hasegawa H McMichael AJ Callan MFC. Human naive CD8 T cells down-regulate expression of the WNT pathway transcription factors lymphoid enhancer binding factor 1 and transcription factor 7 (T cell factor-1) following antigen encounter *in vitro* and *in vivo*1. J Immunol. (2006) 176:1439–46. doi: 10.4049/jimmunol.176.3.1439 16424171

[38] Hogan PG. Calcium–NFAT transcriptional signalling in T cell activation and T cell exhaustion. Cell Calcium. (2017) 63:66–9. doi: 10.1016/j.ceca.2017.01.014 PMC5739523 28153342

[39] Man K Miasari M Shi W Xin A Henstridge DC Preston S. The transcription factor IRF4 is essential for TCR affinity–mediated metabolic programming and clonal expansion of T cells. Nat Immunol. (2013) 14:1155–65. doi: 10.1038/ni.2710 24056747

[40] Yukawa M Jagannathan S Vallabh S Kartashov AV Chen X Weirauch MT. AP-1 activity induced by co-stimulation is required for chromatin opening during T cell activation. J Exp Med. (2019) 217:e20182009. doi: 10.1084/jem.20182009 PMC7037242 31653690

[41] Nurieva RI Liu X Dong C. Molecular mechanisms of T-cell tolerance. Immunol Rev. (2011) 241:133–44. doi: 10.1111/j.1600-065X.2011.01012.x PMC5131796 21488895

[42] Cox MA Harrington LE Zajac AJ. Cytokines and the inception of CD8 T cell responses. Trends Immunol. (2011) 32:180. doi: 10.1016/j.it.2011.01.004 21371940 PMC3074938

[43] Curtsinger JM Mescher MF. Inflammatory cytokines as a third signal for T cell activation. Curr Opin Immunol. (2010) 22:333–40. doi: 10.1016/j.coi.2010.02.013 PMC2891062 20363604

[44] Curtsinger JM Johnson CM Mescher MF. CD8 T cell clonal expansion and development of effector function require prolonged exposure to antigen, costimulation, and signal 3 cytokine 1. J Immunol. (2003) 171:5165–71. doi: 10.4049/jimmunol.171.10.5165 14607916

[45] Joshi NS Cui W Chandele A Lee HK Urso DR Hagman J. Inflammation directs memory precursor and short-lived effector CD8+ T cell fates via the graded expression of T-bet transcription factor. Immunity. (2007) 27:281–95. doi: 10.1016/j.immuni.2007.07.010 PMC2034442 17723218

[46] Plumlee CR Obar JJ Colpitts SL Jellison ER Haining WN Lefrancois L. Early effector CD8 T cells display plasticity in populating the short-lived effector and memory-precursor pools following bacterial or viral infection. Sci Rep. (2015) 5:12264. doi: 10.1038/srep12264 26191658 PMC4507483

[47] Nayar R Schutten E Bautista B Daniels K Prince AL Enos M. Graded levels of IRF4 regulate CD8+ T cell differentiation and expansion, but not attrition, in response to acute virus infection. J Immunol. (2014) 192:5881–93. doi: 10.4049/jimmunol.1303187 PMC4080788 24835398

[48] Dunkle A Dzhagalov I Gordy C He Y-W. Transfer of CD8+ T cell memory using Bcl-2 as a marker. J Immunol. (2013) 190:940–7. doi: 10.4049/jimmunol.1103481 PMC4366938 23269245

[49] Jeannet G Boudousquié C Gardiol N Kang J Huelsken J Held W. Essential role of the Wnt pathway effector Tcf-1 for the establishment of functional CD8 T cell memory. Proc Natl Acad Sci U.S.A. (2010) 107:9777–82. doi: 10.1073/pnas.0914127107 PMC2906901 20457902

[50] Utzschneider DT Charmoy M Chennupati V Pousse L Ferreira DP Calderon-Copete S. T cell factor 1-expressing memory-like CD8+ T cells sustain the immune response to chronic viral infections. Immunity. (2016) 45:415–27. doi: 10.1016/j.immuni.2016.07.021 27533016

[51] Intlekofer AM Takemoto N Wherry EJ Longworth SA Northrup JT Palanivel VR. Effector and memory CD8+ T cell fate coupled by T-bet and eomesodermin. Nat Immunol. (2005) 6:1236–44. doi: 10.1038/ni1268 16273099

[52] Samji T Khanna KM. Understanding memory CD8+ T cells. Immunol Lett. (2017) 185:32–9. doi: 10.1016/j.imlet.2017.02.012 PMC5508124 28274794

[53] Sallusto F Lenig D Förster R Lipp M Lanzavecchia A. Two subsets of memory T lymphocytes with distinct homing potentials and effector functions. Nature. (1999) 401:708–12. doi: 10.1038/44385 10537110

[54] Schenkel JM Masopust D. Tissue-resident memory T cells. Immunity. (2014) 41:886–97. doi: 10.1016/j.immuni.2014.12.007 PMC4276131 25526304

[55] Takada K Wang X Hart G Odumade OA Weinreich MA Hogquist KA. KLF2 is required for trafficking but not quiescence in post-activated T cells. J Immunol. (2011) 186:775–83. doi: 10.4049/jimmunol.1000094 PMC3017213 21160050

[56] Rutishauser RL Martins GA Kalachikov S Chandele A Parish IA Meffre E. Transcriptional repressor Blimp-1 promotes CD8(+) T cell terminal differentiation and represses the acquisition of central memory T cell properties. Immunity. (2009) 31:296–308. doi: 10.1016/j.immuni.2009.05.014 19664941 PMC2783637

[57] Billingsley JM Rajakumar PA Connole MA Salisch NC Adnan S Kuzmichev YV. Characterization of CD8+ T cell differentiation following SIVΔnef vaccination by transcription factor expression profiling. PloS Pathog. (2015) 11:e1004740. doi: 10.1371/journal.ppat.1004740 25768938 PMC4358830

[58] Fonseca R Burn TN Gandolfo LC Devi S Park SL Obers A. Runx3 drives a CD8+ T cell tissue residency program that is absent in CD4+ T cells. Nat Immunol. (2022) 23:1236–45. doi: 10.1038/s41590-022-01273-4 PMC13045866 35882933

[59] Milner JJ Toma C Yu B Zhang K Omilusik K Phan AT. Runx3 programs CD8+ T cell residency in non-lymphoid tissues and tumours. Nature. (2017) 552:253–7. doi: 10.1038/nature24993 PMC5747964 29211713

[60] Mackay LK Minnich M Kragten NAM Liao Y Nota B Seillet C. Hobit and Blimp1 instruct a universal transcriptional program of tissue residency in lymphocytes. Science. (2016) 352:459–63. doi: 10.1126/science.aad2035 27102484

[61] Dolina JS Van Braeckel-Budimir N Thomas GD Salek-Ardakani S. CD8+ T cell exhaustion in cancer. Front Immunol. (2021) 12:715234. doi: 10.3389/fimmu.2021.715234 34354714 PMC8330547

[62] Wherry EJ Kurachi M. Molecular and cellular insights into T cell exhaustion. Nat Rev Immunol. (2015) 15:486–99. doi: 10.1038/nri3862 PMC4889009 26205583

[63] Khan O Giles JR McDonald S Manne S Ngiow SF Patel KP. TOX transcriptionally and epigenetically programs CD8+ T cell exhaustion. Nature. (2019) 571:211–8. doi: 10.1038/s41586-019-1325-x PMC6713202 31207603

[64] Zehn D Thimme R Lugli E de Almeida GP Oxenius A. [amp]]lsquo;Stem-like’ precursors are the fount to sustain persistent CD8+ T cell responses. Nat Immunol. (2022) 23:836–47. doi: 10.1038/s41590-022-01219-w 35624209

[65] Hudson WH Gensheimer J Hashimoto M Wieland A Valanparambil RM Li P. Proliferating transitory T cells with an effector-like transcriptional signature emerge from PD-1+ Stem-like CD8+ T cells during chronic infection. Immunity. (2019) 51:1043–1058.e4. doi: 10.1016/j.immuni.2019.11.002 31810882 PMC6920571

[66] Beltra J-C Manne S Abdel-Hakeem MS Kurachi M Giles JR Chen Z. Developmental relationships of four exhausted CD8+ T cell subsets reveals underlying transcriptional and epigenetic landscape control mechanisms. Immunity. (2020) 52:825–841.e8. doi: 10.1016/j.immuni.2020.04.014 32396847 PMC8360766

[67] Nah J Seong RH. Krüppel-like factor 4 regulates the cytolytic effector function of exhausted CD8 T cells. Sci Adv. (2022) 8:eadc9346. doi: 10.1126/sciadv.adc9346 36427304 PMC9699681

[68] Park J Hsueh P-C Li Z Ho P-C. Microenvironment-driven metabolic adaptations guiding CD8+ T cell anti-tumor immunity. Immunity. (2023) 56:32–42. doi: 10.1016/j.immuni.2022.12.008 36630916

[69] Bannoud N Dalotto-Moreno T Kindgard L García PA Blidner AG Mariño KV. Hypoxia supports differentiation of terminally exhausted CD8 T cells. Front Immunol. (2021) 12:660944. doi: 10.3389/fimmu.2021.660944 34025660 PMC8137905

[70] Chen GM Chen C Das RK Gao P Chen C-H Bandyopadhyay S. Integrative bulk and single-cell profiling of premanufacture T-cell populations reveals factors mediating long-term persistence of CAR T-cell therapy. Cancer Discovery. (2021) 11:2186–99. doi: 10.1158/2159-8290.CD-20-1677 PMC8419030 33820778

[71] Li P Burke S Wang J Chen X Ortiz M Lee S-C. Reprogramming of T cells to natural killer–like cells upon bcl11b deletion. Science. (2010) 329:85–9. doi: 10.1126/science.1188063 PMC3628452 20538915

[72] Kim K Park S Park SY Kim G Park SM Cho J-W. Single-cell transcriptome analysis reveals TOX as a promoting factor for T cell exhaustion and a predictor for anti-PD-1 responses in human cancer. Genome Med. (2020) 12:22. doi: 10.1186/s13073-020-00722-9 32111241 PMC7048139

[73] Chen J López-Moyado IF Seo H Lio C-WJ Hempleman LJ Sekiya T. NR4A transcription factors limit CAR T cell function in solid tumours. Nature. (2019) 567:530–4. doi: 10.1038/s41586-019-0985-x PMC6546093 30814732

[74] Zhu Z Lou G Teng X-L Wang H Luo Y Shi W. FOXP1 and KLF2 reciprocally regulate checkpoints of stem-like to effector transition in CAR T cells. Nat Immunol. (2024) 25:117–28. doi: 10.1038/s41590-023-01685-w PMC10841689 38012417

[75] Lachmann A Giorgi FM Lopez G Califano A. ARACNe-AP: gene network reverse engineering through adaptive partitioning inference of mutual information. Bioinformatics. (2016) 32:2233–5. doi: 10.1093/bioinformatics/btw216 PMC4937200 27153652

[76] Huynh-Thu VA Irrthum A Wehenkel L Geurts P. Inferring regulatory networks from expression data using tree-based methods. PloS One. (2010) 5:e12776. doi: 10.1371/journal.pone.0012776 20927193 PMC2946910

[77] Langfelder P Horvath S. WGCNA: an R package for weighted correlation network analysis. BMC Bioinf. (2008) 9:559. doi: 10.1186/1471-2105-9-559 PMC2631488 19114008

[78] Heinz S Benner C Spann N Bertolino E Lin YC Laslo P. Simple combinations of lineage-determining transcription factors prime cis-regulatory elements required for macrophage and B cell identities. Mol Cell. (2010) 38:576–89. doi: 10.1016/j.molcel.2010.05.004 PMC2898526 20513432

[79] Garcia-Alonso L Holland CH Ibrahim MM Turei D Saez-Rodriguez J. Benchmark and integration of resources for the estimation of human transcription factor activities. Genome Res. (2019) 29:1363–75. doi: 10.1101/gr.240663.118 PMC6673718 31340985

[80] Cornwell M Vangala M Taing L Herbert Z Köster J Li B. VIPER: Visualization Pipeline for RNA-seq, a Snakemake workflow for efficient and complete RNA-seq analysis. BMC Bioinf. (2018) 19:135. doi: 10.1186/s12859-018-2139-9 PMC5897949 29649993

[81] Aibar S González-Blas CB Moerman T Huynh-Thu VA Imrichova H Hulselmans G. SCENIC: single-cell regulatory network inference and clustering. Nat Methods. (2017) 14:1083–6. doi: 10.1038/nmeth.4463 PMC5937676 28991892

[82] Lefebvre C Rajbhandari P Alvarez MJ Bandaru P Lim WK Sato M. A human B-cell interactome identifies MYB and FOXM1 as master regulators of proliferation in germinal centers. Mol Syst Biol. (2010) 6:377. doi: 10.1038/msb.2010.31 20531406 PMC2913282

[83] Kamimoto K Stringa B Hoffmann CM Jindal K Solnica-Krezel L Morris SA. Dissecting cell identity via network inference and in silico gene perturbation. Nature. (2023) 614:742–51. doi: 10.1038/s41586-022-05688-9 PMC9946838 36755098

[84] Jin X Cai Y Xue G Que J Cheng R Yang Y. Identification of shared characteristics in tumor-infiltrating T cells across 15 cancers. Mol Ther Nucleic Acids. (2023) 32:189–202c. doi: 10.1016/j.omtn.2023.03.007 PMC10122023 37096165

[85] Qian J Olbrecht S Boeckx B Vos H Laoui D Etlioglu E. A pan-cancer blueprint of the heterogeneous tumor microenvironment revealed by single-cell profiling. Cell Res. (2020) 30:745–62. doi: 10.1038/s41422-020-0355-0 PMC7608385 32561858

[86] Melenhorst JJ Chen GM Wang M Porter DL Chen C Collins MA. Decade-long leukaemia remissions with persistence of CD4+ CAR T cells. Nature. (2022) 602:503–9. doi: 10.1038/s41586-021-04390-6 PMC9166916 35110735

[87] Zhou P Shi H Huang H Sun X Yuan S Chapman NM. Single-cell CRISPR screens in *vivo* map T cell fate regulomes in cancer. Nature. (2023) 624:154–63. doi: 10.1038/s41586-023-06733-x PMC10700132 37968405

[88] Schmidt R Steinhart Z Layeghi M Freimer JW Bueno R Nguyen VQ. CRISPR activation and interference screens decode stimulation responses in primary human T cells. Science. (2022) 375:eabj4008. doi: 10.1126/science.abj4008 35113687 PMC9307090

[89] Obradovic A Ager C Turunen M Nirschl T Khosravi-Maharlooei M Iuga A. Systematic elucidation and pharmacological targeting of tumor-infiltrating regulatory T cell master regulators. Cancer Cell. (2023) 41:933–949.e11. doi: 10.1016/j.ccell.2023.04.003 37116491 PMC10193511

[90] Liu Y Zhou N Zhou L Wang J Zhou Y Zhang T. IL-2 regulates tumor-reactive CD8+ T cell exhaustion by activating the aryl hydrocarbon receptor. Nat Immunol. (2021) 22:358–69. doi: 10.1038/s41590-020-00850-9 33432230

[91] Giuffrida L Sek K Henderson MA House IG Lai J Chen AXY. IL-15 preconditioning augments CAR T cell responses to checkpoint blockade for improved treatment of solid tumors. Mol Ther. (2020) 28:2379–93. doi: 10.1016/j.ymthe.2020.07.018 PMC7647667 32735774

[92] Chi H. Regulation and function of mTOR signalling in T cell fate decisions. Nat Rev Immunol. (2012) 12:325–38. doi: 10.1038/nri3198 PMC3417069 22517423

[93] Sinclair LV Finlay D Feijoo C Cornish GH Gray A Ager A. Phosphatidylinositol-3-OH kinase and nutrient-sensing mTOR pathways control T lymphocyte trafficking. Nat Immunol. (2008) 9:513–21. doi: 10.1038/ni.1603 PMC2857321 18391955

[94] Wang Z Zhou G Risu N Fu J Zou Y Tang J. Lenalidomide enhances CAR-T cell activity against solid tumor cells. Cell Transplant. (2020) 29:963689720920825. doi: 10.1177/0963689720920825 32967454 PMC7784582

[95] Dai Z Sezin T Chang Y Lee EY Wang EHC Christiano AM. Induction of T cell exhaustion by JAK1/3 inhibition in the treatment of alopecia areata. Front Immunol. (2022) 13:955038. doi: 10.3389/fimmu.2022.955038 36203601 PMC9531018

[96] Geiger R Rieckmann JC Wolf T Basso C Feng Y Fuhrer T. L-arginine modulates T cell metabolism and enhances survival and anti-tumor activity. Cell. (2016) 167:829–842.e13. doi: 10.1016/j.cell.2016.09.031 27745970 PMC5075284

[97] Chisolm DA Savic D Moore AJ Ballesteros-Tato A León B Crossman DK. CCCTC-binding factor translates interleukin 2- and α-ketoglutarate-sensitive metabolic changes in T cells into context-dependent gene programs. Immunity. (2017) 47:251–267.e7. doi: 10.1016/j.immuni.2017.07.015 28813658 PMC5654635

[98] Jinek M Chylinski K Fonfara I Hauer M Doudna JA Charpentier E. A programmable dual-RNA-guided DNA endonuclease in adaptive bacterial immunity. Science. (2012) 337:816–21. doi: 10.1126/science.1225829 PMC6286148 22745249

[99] Javaid D Ganie SY Hajam YA Reshi MS. CRISPR/Cas9 system: a reliable and facile genome editing tool in modern biology. Mol Biol Rep. (2022) 49:12133–50. doi: 10.1007/s11033-022-07880-6 PMC9420241 36030476

[100] Adli M. The CRISPR tool kit for genome editing and beyond. Nat Commun. (2018) 9:1911. doi: 10.1038/s41467-018-04252-2 29765029 PMC5953931

[101] Qi LS Larson MH Gilbert LA Doudna JA Weissman JS Arkin AP. Repurposing CRISPR as an RNA-guided platform for sequence-specific control of gene expression. Cell. (2013) 152:1173–83. doi: 10.1016/j.cell.2013.02.022 PMC3664290 23452860

[102] Mocellin S Provenzano M. RNA interference: learning gene knock-down from cell physiology. J Transl Med. (2004) 2:39. doi: 10.1186/1479-5876-2-39 15555080 PMC534783

[103] Elbashir SM Harborth J Lendeckel W Yalcin A Weber K Tuschl T. Duplexes of 21-nucleotide RNAs mediate RNA interference in cultured mammalian cells. Nature. (2001) 411:494–8. doi: 10.1038/35078107 11373684

[104] Paddison PJ Caudy AA Hannon GJ. Stable suppression of gene expression by RNAi in mammalian cells. Proc Natl Acad Sci U.S.A. (2002) 99:1443–8. doi: 10.1073/pnas.032652399 PMC122210 11818553

[105] Moore CB Guthrie EH Huang MT-H Taxman DJ. Short hairpin RNA (shRNA): design, delivery, and assessment of gene knockdown. Methods Mol Biol. (2010) 629:141–58. doi: 10.1007/978-1-60761-657-3_10 PMC3679364 20387148

[106] McLellan MA Rosenthal NA Pinto AR. Cre-loxP-mediated recombination: general principles and experimental considerations. Curr Protoc Mouse Biol. (2017) 7:1–12. doi: 10.1002/cpmo.22 28252198

[107] Gu H Zou Y-R Rajewsky K. Independent control of immunoglobulin switch recombination at individual switch regions evidenced through Cre-loxP-mediated gene targeting. Cell. (1993) 73:1155–64. doi: 10.1016/0092-8674(93)90644-6 8513499

[108] Lee PP Fitzpatrick DR Beard C Jessup HK Lehar S Makar KW. A critical role for dnmt1 and DNA methylation in T cell development, function, and survival. Immunity. (2001) 15:763–74. doi: 10.1016/S1074-7613(01)00227-8 11728338

[109] Shi J Petrie HT. Activation kinetics and off-target effects of thymus-initiated cre transgenes. PloS One. (2012) 7:e46590. doi: 10.1371/journal.pone.0046590 23049709 PMC3462198

[110] Jacob J Baltimore D. Modelling T-cell memory by genetic marking of memory T cells in *vivo* . Nature. (1999) 399:593–7. doi: 10.1038/21208 10376601

[111] Bulcha JT Wang Y Ma H Tai PWL Gao G. Viral vector platforms within the gene therapy landscape. Sig Transduct Target Ther. (2021) 6:1–24. doi: 10.1038/s41392-021-00487-6 PMC7868676 33558455

[112] Lin S Staahl BT Alla RK Doudna JA. Enhanced homology-directed human genome engineering by controlled timing of CRISPR/Cas9 delivery. eLife. (2014) 3:e04766. doi: 10.7554/eLife.04766 25497837 PMC4383097

[113] Zhao Y Zheng Z Cohen CJ Gattinoni L Palmer DC Restifo NP. High-efficiency transfection of primary human and mouse T lymphocytes using RNA electroporation. Mol Ther. (2006) 13:151–9. doi: 10.1016/j.ymthe.2005.07.688 PMC1473967 16140584

[114] Chong ZX Yeap SK Ho WY. Transfection types, methods and strategies: a technical review. PeerJ. (2021) 9:e11165. doi: 10.7717/peerj.11165 33976969 PMC8067914

[115] Konermann S Brigham MD Trevino AE Joung J Abudayyeh OO Barcena C. Genome-scale transcriptional activation by an engineered CRISPR-Cas9 complex. Nature. (2015) 517:583–8. doi: 10.1038/nature14136 PMC4420636 25494202

[116] Pan M-G Xiong Y Chen F. NFAT gene family in inflammation and cancer. Curr Mol Med. (2013) 13:543–54. doi: 10.2174/1566524011313040007 PMC3694398 22950383

[117] Go WY Liu X Roti MA Liu F Ho SN. NFAT5/TonEBP mutant mice define osmotic stress as a critical feature of the lymphoid microenvironment. Proc Natl Acad Sci U.S.A. (2004) 101:10673–8. doi: 10.1073/pnas.0403139101 PMC489993 15247420

[118] Hogan PG Chen L Nardone J Rao A. Transcriptional regulation by calcium, calcineurin, and NFAT. Genes Dev. (2003) 17:2205–32. doi: 10.1101/gad.1102703 12975316

[119] Rooney JW Sun YL Glimcher LH Hoey T. Novel NFAT sites that mediate activation of the interleukin-2 promoter in response to T-cell receptor stimulation. Mol Cell Biol. (1995) 15:6299–310. doi: 10.1128/MCB.15.11.6299 PMC230882 7565783

[120] Martinez GJ Pereira RM Äijö T Kim EY Marangoni F Pipkin ME. The transcription factor NFAT promotes exhaustion of activated CD8^+^ T cells. Immunity. (2015) 42:265–78. doi: 10.1016/j.immuni.2015.01.006 PMC4346317 25680272

[121] Tillé L Cropp D Charmoy M Reichenbach P Andreatta M Wyss T. Activation of the transcription factor NFAT5 in the tumor microenvironment enforces CD8+ T cell exhaustion. Nat Immunol. (2023) 24:1645–53. doi: 10.1038/s41590-023-01614-x 37709986

[122] Heim L Friedrich J Engelhardt M Trufa DI Geppert CI Rieker RJ. NFATc1 promotes antitumoral effector functions and memory CD8+ T-cell differentiation during non-small cell lung cancer development. Cancer Res. (2018) 78:3619–33. doi: 10.1158/0008-5472.CAN-17-3297 29691251

[123] Aliahmad P Seksenyan A Kaye J. The many roles of TOX in the immune system. Curr Opin Immunol. (2012) 24:173–7. doi: 10.1016/j.coi.2011.12.001 PMC3319641 22209117

[124] Wang X He Q Shen H Xia A Tian W Yu W. TOX promotes the exhaustion of antitumor CD8+ T cells by preventing PD1 degradation in hepatocellular carcinoma. J Hepatol. (2019) 71:731–41. doi: 10.1016/j.jhep.2019.05.015 31173813

[125] Seo H Chen J González-Avalos E Samaniego-Castruita D Das A Wang YH. TOX and TOX2 transcription factors cooperate with NR4A transcription factors to impose CD8+ T cell exhaustion. Proc Natl Acad Sci U.S.A. (2019) 116:12410–5. doi: 10.1073/pnas.1905675116 PMC6589758 31152140

[126] Scott AC Dündar F Zumbo P Chandran SS Klebanoff CA Shakiba M. TOX is a critical regulator of tumour-specific T cell differentiation. Nature. (2019) 571:270–4. doi: 10.1038/s41586-019-1324-y PMC7698992 31207604

[127] Alfei F Kanev K Hofmann M Wu M Ghoneim HE Roelli P. TOX reinforces the phenotype and longevity of exhausted T cells in chronic viral infection. Nature. (2019) 571:265–9. doi: 10.1038/s41586-019-1326-9 31207605

[128] Yao C Sun H-W Lacey NE Ji Y Moseman EA Shih H-Y. Single-cell RNA-seq reveals TOX as a key regulator of CD8+ T cell persistence in chronic infection. Nat Immunol. (2019) 20:890–901. doi: 10.1038/s41590-019-0403-4 31209400 PMC6588409

[129] Collins SM Alexander KA Lundh S Dimitri AJ Zhang Z Good CR. TOX2 coordinates with TET2 to positively regulate central memory differentiation in human CAR T cells. Sci Adv. (2023) 9:eadh2605. doi: 10.1126/sciadv.adh2605 37467321 PMC10355826

[130] Odagiu L May J Boulet S Baldwin TA Labrecque N. Role of the orphan nuclear receptor NR4A family in T-cell biology. Front Endocrinol. (2021) 11:624122. doi: 10.3389/fendo.2020.624122 PMC7883379 33597928

[131] Odagiu L Boulet S Maurice De Sousa D Daudelin J-F Nicolas S Labrecque N. Early programming of CD8 ^+^ T cell response by the orphan nuclear receptor NR4A3. Proc Natl Acad Sci U.S.A. (2020) 117:24392–402. doi: 10.1073/pnas.2007224117 PMC7533658 32913051

[132] Hibino S Chikuma S Kondo T Ito M Nakatsukasa H Omata-Mise S. Inhibition of nr4a receptors enhances antitumor immunity by breaking treg-mediated immune tolerance. Cancer Res. (2018) 78:3027–40. doi: 10.1158/0008-5472.CAN-17-3102 29559474

[133] Hiwa R Nielsen HV Mueller JL Mandla R Zikherman J. NR4A family members regulate T cell tolerance to preserve immune homeostasis and suppress autoimmunity. JCI Insight. (2021) 6:e151005. doi: 10.1172/jci.insight.151005 34343134 PMC8492309

[134] Hu QN Suen AYW Henao Caviedes LM Baldwin TA. Nur77 regulates nondeletional mechanisms of tolerance in T cells. J Immunol. (2017) 199:3147–57. doi: 10.4049/jimmunol.1701085 28947542

[135] Liu X Wang Y Lu H Li J Yan X Xiao M. Genome-wide analysis identifies NR4A1 as a key mediator of T cell dysfunction. Nature. (2019) 567:525–9. doi: 10.1038/s41586-019-0979-8 PMC6507425 30814730

[136] Shapiro-Shelef M Calame K. Regulation of plasma-cell development. Nat Rev Immunol. (2005) 5:230–42. doi: 10.1038/nri1572 15738953

[137] Kallies A Xin A Belz GT Nutt SL. Blimp-1 transcription factor is required for the differentiation of effector CD8+ T cells and memory responses. Immunity. (2009) 31:283–95. doi: 10.1016/j.immuni.2009.06.021 19664942

[138] Ji Y Pos Z Rao M Klebanoff CA Yu Z Sukumar M. Repression of the DNA-binding inhibitor Id3 by Blimp-1 limits CD8+ T cell memory formation. Nat Immunol. (2011) 12:1230–7. doi: 10.1038/ni.2153 PMC3226770 22057288

[139] Shin H Blackburn SD Intlekofer AM Kao C Angelosanto JM Reiner SL. A role for the transcriptional repressor blimp-1 in CD8+ T cell exhaustion during chronic viral infection. Immunity. (2009) 31:309–20. doi: 10.1016/j.immuni.2009.06.019 PMC2747257 19664943

[140] Sun Q Cai D Liu D Zhao X Li R Xu W. BCL6 promotes a stem-like CD8+ T cell program in cancer via antagonizing BLIMP1. Sci Immunol. (2023) 8:eadh1306. doi: 10.1126/sciimmunol.adh1306 37862431

[141] Jung I-Y Narayan V McDonald S Rech AJ Bartoszek R Hong G. BLIMP1 and NR4A3 transcription factors reciprocally regulate antitumor CAR T cell stemness and exhaustion. Sci Transl Med. (2022) 14:eabn7336. doi: 10.1126/scitranslmed.abn7336 36350986 PMC10257143

[142] Yoshikawa T Wu Z Inoue S Kasuya H Matsushita H Takahashi Y. Genetic ablation of PRDM1 in antitumor T cells enhances therapeutic efficacy of adoptive immunotherapy. Blood. (2022) 139:2156–72. doi: 10.1182/blood.2021012714 34861037

[143] Azadbakht M Sayadmanesh A Nazer N Ahmadi A Hemmati S Mohammadzade H. CRISPRi-mediated knock-down of PRDM1/BLIMP1 programs central memory differentiation in ex vivo-expanded human T cells. Bioimpacts. (2022) 12:337–47. doi: 10.34172/bi.2021.23522 PMC9376159 35975204

[144] Papavassiliou AG Musti AM. The Multifaceted Output of c-Jun Biological Activity: Focus at the Junction of CD8 T Cell Activation and Exhaustion. Cells. (2020) 9:2470. doi: 10.3390/cells9112470 33202877 PMC7697663

[145] Macián F García-Rodríguez C Rao A. Gene expression elicited by NFAT in the presence or absence of cooperative recruitment of Fos and Jun. EMBO J. (2000) 19:4783–95. doi: 10.1093/emboj/19.17.4783 PMC302068 10970869

[146] Lynn RC Weber EW Sotillo E Gennert D Xu P Good Z. c-Jun overexpression in CAR T cells induces exhaustion resistance. Nature. (2019) 576:293–300. doi: 10.1038/s41586-019-1805-z 31802004 PMC6944329

[147] Hussein MS Li Q Mao R Peng Y He Y. TCR T cells overexpressing c-Jun have better functionality with improved tumor infiltration and persistence in hepatocellular carcinoma. Front Immunol. (2023) 14:1114770. doi: 10.3389/fimmu.2023.1114770 37215108 PMC10192869

[148] Lin W-HW Nish SA Yen B Chen Y-H Adams WC Kratchmarov R. CD8+ T lymphocyte self-renewal during effector cell determination. Cell Rep. (2016) 17:1773–82. doi: 10.1016/j.celrep.2016.10.032 PMC5108530 27829149

[149] Ladle BH Li K-P Phillips MJ Pucsek AB Haile A Powell JD. *De novo* DNA methylation by DNA methyltransferase 3a controls early effector CD8+ T-cell fate decisions following activation. Proc Natl Acad Sci U.S.A. (2016) 113:10631–6. doi: 10.1073/pnas.1524490113 PMC5035851 27582468

[150] Tiemessen MM Baert MRM Kok L van Eggermond MCJA van den Elsen PJ Arens R. T cell factor 1 represses CD8+ Effector T cell formation and function. J Immunol. (2014) 193:5480–7. doi: 10.4049/jimmunol.1303417 25355919

[151] Utzschneider DT Gabriel SS Chisanga D Gloury R Gubser PM Vasanthakumar A. Early precursor T cells establish and propagate T cell exhaustion in chronic infection. Nat Immunol. (2020) 21:1256–66. doi: 10.1038/s41590-020-0760-z 32839610

[152] Miller BC Sen DR Abosy RA Bi K Virkud YV LaFleur MW. Subsets of exhausted CD8+ T cells differentially mediate tumor control and respond to checkpoint blockade. Nat Immunol. (2019) 20:326–36. doi: 10.1038/s41590-019-0312-6 PMC6673650 30778252

[153] Shan Q Hu S Chen X Danahy DB Badovinac VP Zang C. Ectopic Tcf1 expression instills a stem-like program in exhausted CD8+ T cells to enhance viral and tumor immunity. Cell Mol Immunol. (2021) 18:1262–77. doi: 10.1038/s41423-020-0436-5 PMC8093427 32341523

[154] Yang CY Best JA Knell J Yang E Sheridan AD Jesionek AK. The transcriptional regulators Id2 and Id3 control the formation of distinct memory CD8+ T cell subsets. Nat Immunol. (2011) 12(12):1221–9. doi: 10.1038/ni.2158 PMC3872000 22057289

[155] Jin Y Hu P Sun H Yang C Zhai J Wang Y. Expression of Id3 represses exhaustion of anti-tumor CD8 T cells in liver cancer. Mol Immunol. (2022) 144:117–26. doi: 10.1016/j.molimm.2022.02.005 35219016

[156] Tsui C Kretschmer L Rapelius S Gabriel SS Chisanga D Knöpper K. MYB orchestrates T cell exhaustion and response to checkpoint inhibition. Nature. (2022) 609:354–60. doi: 10.1038/s41586-022-05105-1 PMC9452299 35978192

[157] Chen Z Stelekati E Kurachi M Yu S Cai Z Manne S. MiR-150 regulates memory CD8 T cell differentiation via c-Myb. Cell Rep. (2017) 20:2584–97. doi: 10.1016/j.celrep.2017.08.060 PMC5611819 28903040

[158] Allen RD Bender TP Siu G. c-Myb is essential for early T cell development. Genes Dev. (1999) 13:1073–8. doi: 10.1101/gad.13.9.1073 PMC316940 10323859

[159] Bender TP Kremer CS Kraus M Buch T Rajewsky K. Critical functions for c-Myb at three checkpoints during thymocyte development. Nat Immunol. (2004) 5:721–9. doi: 10.1038/ni1085 15195090

[160] Gautam S Fioravanti J Zhu W Le Gall JB Brohawn P Lacey NE. The transcription factor c-Myb regulates CD8+ T cell stemness and antitumor immunity. Nat Immunol. (2019) 20:337–49. doi: 10.1038/s41590-018-0311-z PMC6489499 30778251

[161] Utzschneider DT Delpoux A Wieland D Huang X Lai C-Y Hofmann M. Active maintenance of T cell memory in acute and chronic viral infection depends on continuous expression of FOXO1. Cell Rep. (2018) 22:3454–67. doi: 10.1016/j.celrep.2018.03.020 PMC5942184 29590615

[162] Delpoux A Marcel N Hess Michelini R Katayama CD Allison KA Glass CK. FOXO1 constrains activation and regulates senescence in CD8 T cells. Cell Rep. (2021) 34:108674. doi: 10.1016/j.celrep.2020.108674 33503413

[163] Delpoux A Lai C-Y Hedrick SM Doedens AL. FOXO1 opposition of CD8+ T cell effector programming confers early memory properties and phenotypic diversity. Proc Natl Acad Sci. (2017) 114:E8865–74. doi: 10.1073/pnas.1618916114 PMC5651728 28973925

[164] Doan AE Mueller KP Chen AY Rouin GT Chen Y Daniel B. FOXO1 is a master regulator of memory programming in CAR T cells. Nature. (2024) 629:211–8. doi: 10.1038/s41586-024-07300-8 PMC11062920 38600391

[165] Chan JD Scheffler CM Munoz I Sek K Lee JN Huang Y-K. FOXO1 enhances CAR T cell stemness, metabolic fitness and efficacy. Nature. (2024) 629:201–10. doi: 10.1038/s41586-024-07242-1 PMC11062918 38600376

[166] Woolf E Xiao C Fainaru O Lotem J Rosen D Negreanu V. Runx3 and Runx1 are required for CD8 T cell development during thymopoiesis. Proc Natl Acad Sci U.S.A. (2003) 100:7731–6. doi: 10.1073/pnas.1232420100 PMC164656 12796513

[167] Wang D Diao H Getzler AJ Rogal W Frederick MA Milner J. The transcription factor runx3 establishes chromatin accessibility of cis-regulatory landscapes that drive memory cytotoxic T lymphocyte formation. Immunity. (2018) 48:659–674.e6. doi: 10.1016/j.immuni.2018.03.028 29669249 PMC6750808

[168] Shan Q Zeng Z Xing S Li F Hartwig SM Gullicksrud JA. Runx3 guards cytotoxic CD8+ effector T cells against deviation towards TFH cell lineage. Nat Immunol. (2017) 18:931–9. doi: 10.1038/ni.3773 PMC5564218 28604718

[169] Strobl J Pandey RV Krausgruber T Bayer N Kleissl L Reininger B. Long-term skin-resident memory T cells proliferate in situ and are involved in human graft-versus-host disease. Sci Transl Med. (2020) 12:eabb7028. doi: 10.1126/scitranslmed.abb7028 33208504 PMC7615006

[170] Song Q Shang J Zhang C Chen J Zhang L Wu X. Transcription factor RUNX3 promotes CD8+ T cell recruitment by CCL3 and CCL20 in lung adenocarcinoma immune microenvironment. J Cell Biochem. (2020) 121:3208–20. doi: 10.1002/jcb.29587 31898342

[171] Zhu X Li W Gao J Shen J Xu Y Zhang C. RUNX3 improves CAR-T cell phenotype and reduces cytokine release while maintaining CAR-T function. Med Oncol. (2023) 40:89. doi: 10.1007/s12032-022-01913-7 36735165

[172] Wang Y Zhang H Du G Luo H Su J Sun Y. Enforced expression of Runx3 improved CAR-T cell potency in solid tumor via enhancing resistance to activation-induced cell death. Mol Ther. (2023) 31:701–14. doi: 10.1016/j.ymthe.2022.12.009 PMC10014350 36523165

[173] Tang J Sheng J Zhang Q Ji Y Wang X Zhang J. Runx3-overexpression cooperates with ex vivo AKT inhibition to generate receptor-engineered T cells with better persistence, tumor-residency, and antitumor ability. J Immunother Cancer. (2023) 11:e006119. doi: 10.1136/jitc-2022-006119 36849200 PMC9972435

[174] Tissue-resident memory CAR T cells with stem-like characteristics display enhanced efficacy against solid and liquid tumors - PMC . Available online at: https://www.ncbi.nlm.nih.gov/pmc/articles/PMC10313923/ (Accessed August 6, 2024). 10.1016/j.xcrm.2023.101053 PMC10313923 37224816

[175] Ziello JE Jovin IS Huang Y. Hypoxia-inducible factor (HIF)-1 regulatory pathway and its potential for therapeutic intervention in Malignancy and ischemia. Yale J Biol Med. (2007) 80:51. doi: 10.3390/cancers14246054 18160990 PMC2140184

[176] Li Y Zhao L Li X-F. Hypoxia and the tumor microenvironment. Technol Cancer Res Treat. (2021) 20:15330338211036304. doi: 10.1177/15330338211036304 34350796 PMC8358492

[177] Doedens AL Phan AT Stradner MH Fujimoto JK Nguyen JV Yang E. Hypoxia-inducible factors enhance the effector responses of CD8+ T cells to persistent antigen. Nat Immunol. (2013) 14:1173–82. doi: 10.1038/ni.2714 PMC3977965 24076634

[178] Liikanen I Lauhan C Quon S Omilusik K Phan AT Bartrolí LB. Hypoxia-inducible factor activity promotes antitumor effector function and tissue residency by CD8+ T cells. J Clin Invest. (2021) 131(7):e143729. doi: 10.1172/JCI143729 33792560 PMC8011896

[179] Palazon A Tyrakis PA Macias D Veliça P Rundqvist H Fitzpatrick S. An HIF-1α/VEGF-A axis in cytotoxic T cells regulates tumor progression. Cancer Cell. (2017) 32:669–683.e5. doi: 10.1016/j.ccell.2017.10.003 29136509 PMC5691891

[180] Veliça P Cunha PP Vojnovic N Foskolou IP Bargiela D Gojkovic M. Modified hypoxia-inducible factor expression in CD8+ T cells increases antitumor efficacy. Cancer Immunol Res. (2021) 9:401–14. doi: 10.1158/2326-6066.CIR-20-0561 PMC7611205 33602720

[181] Punta M Coggill PC Eberhardt RY Mistry J Tate J Boursnell C. The Pfam protein families database. Nucleic Acids Res. (2012) 40:D290–301. doi: 10.1093/nar/gkr1065 PMC3245129 22127870

[182] Ataide MA Komander K Knöpper K Peters AE Wu H Eickhoff S. BATF3 programs CD8+ T cell memory. Nat Immunol. (2020) 21:1397–407. doi: 10.1038/s41590-020-0786-2 32989328

[183] Qiu Z Khairallah C Romanov G Sheridan BS. Batf3 expression by CD8 T cells critically regulates the development of memory populations. J Immunol. (2020) 205:901. doi: 10.4049/jimmunol.2000228 32669309 PMC7539233

[184] McCutcheon SR Swartz AM Brown MC Barrera A McRoberts Amador C Siklenka K. Transcriptional and epigenetic regulators of human CD8+ T cell function identified through orthogonal CRISPR screens. Nat Genet. (2023) 55:2211–23. doi: 10.1038/s41588-023-01554-0 PMC10703699 37945901

[185] Jain N Zhao Z Feucht J Koche R Iyer A Dobrin A. TET2 guards against unchecked BATF3-induced CAR T cell expansion. Nature. (2023) 615:315–22. doi: 10.1038/s41586-022-05692-z PMC10511001 36755094

[186] Tsuda SM Diao H Getzler A Milner J Goldrath AW Crotty S. Ets1 governs the differential formation of circulating and tissue-resident memory CD8 T cells. J Immunol. (2021) 206:98. doi: 10.4049/jimmunol.206.Supp.98.56

[187] Mouly E Chemin K Nguyen HV Chopin M Mesnard L Leite-de-Moraes M. The Ets-1 transcription factor controls the development and function of natural regulatory T cells. J Exp Med. (2010) 207:2113–25. doi: 10.1084/jem.20092153 PMC2947068 20855499

[188] Muthusamy N Barton K Leiden JM. Defective activation and survival of T cells lacking the Ets-1 transcription factor. Nature. (1995) 377:639–42. doi: 10.1038/377639a0 7566177

[189] Grenningloh R Tai T-S Frahm N Hongo TC Chicoine AT Brander C. Ets-1 maintains IL-7 receptor expression in peripheral T cells. J Immunol. (2011) 186:969–76. doi: 10.4049/jimmunol.1002099 PMC3074256 21148801

[190] Chen Z Arai E Khan O Zhang Z Ngiow SF He Y. *In vivo* CD8+ T cell CRISPR screening reveals control by Fli1 in infection and cancer. Cell. (2021) 184:1262–1280.e22. doi: 10.1016/j.cell.2021.02.019 33636129 PMC8054351

[191] Grusdat M McIlwain DR Xu HC Pozdeev VI Knievel J Crome SQ. IRF4 and BATF are critical for CD8^+^ T-cell function following infection with LCMV. Cell Death Differ. (2014) 21:1050–60. doi: 10.1038/cdd.2014.19 PMC4207473 24531538

[192] Godec J Cowley GS Barnitz RA Alkan O Root DE Sharpe AH. Inducible RNAi in *vivo* reveals that the transcription factor BATF is required to initiate but not maintain CD8+ T-cell effector differentiation. Proc Natl Acad Sci U.S.A. (2015) 112:512–7. doi: 10.1073/pnas.1413291112 PMC4299213 25548173

[193] Kurachi M Barnitz RA Yosef N Odorizzi PM DiIorio MA Lemieux ME. The transcription factor BATF operates as an essential differentiation checkpoint in early effector CD8+ T cells. Nat Immunol. (2014) 15:373–83. doi: 10.1038/ni.2834 PMC4000237 24584090

[194] Zebley CC Brown C Mi T Fan Y Alli S Boi S. CD19-CAR T cells undergo exhaustion DNA methylation programming in patients with acute lymphoblastic leukemia. Cell Rep. (2021) 37:110079. doi: 10.1016/j.celrep.2021.110079 34852226 PMC8800370

[195] Jiang P Zhang Z Hu Y Liang Z Han Y Li X. Single-cell ATAC-seq maps the comprehensive and dynamic chromatin accessibility landscape of CAR-T cell dysfunction. Leukemia. (2022) 36:2656–68. doi: 10.1038/s41375-022-01676-0 35962059

[196] Kagoya Y Nakatsugawa M Yamashita Y Ochi T Guo T Anczurowski M. BET bromodomain inhibition enhances T cell persistence and function in adoptive immunotherapy models. J Clin Invest. (2016) 126:3479–94. doi: 10.1172/JCI86437 PMC5004946 27548527

[197] Zhang X Zhang C Qiao M Cheng C Tang N Lu S. Depletion of BATF in CAR-T cells enhances antitumor activity by inducing resistance against exhaustion and formation of central memory cells. Cancer Cell. (2022) 40:1407–1422.e7. doi: 10.1016/j.ccell.2022.09.013 36240777

[198] Seo H González-Avalos E Zhang W Ramchandani P Yang C Lio C-WJ. BATF and IRF4 cooperate to counter exhaustion in tumor-infiltrating CAR T cells. Nat Immunol. (2021) 22:983–95. doi: 10.1038/s41590-021-00964-8 PMC8319109 34282330

[199] Chen Y Zander RA Wu X Schauder DM Kasmani MY Shen J. BATF regulates progenitor to cytolytic effector CD8+ T cell transition during chronic viral infection. Nat Immunol. (2021) 22:996–1007. doi: 10.1038/s41590-021-00965-7 34282329 PMC9258987

[200] Man K Gabriel SS Liao Y Gloury R Preston S Henstridge DC. Transcription factor IRF4 promotes CD8+ T cell exhaustion and limits the development of memory-like T cells during chronic infection. Immunity. (2017) 47:1129–1141.e5. doi: 10.1016/j.immuni.2017.11.021 29246443

[201] Yan H Dai Y Zhang X Zhang H Xiao X Fu J. The transcription factor IRF4 determines the anti-tumor immunity of CD8+ T cells. iScience. (2023) 26:108087. doi: 10.1016/j.isci.2023.108087 37860697 PMC10583049

[202] Yu A Fu J Yin Z Yan H Xiao X Zou D. Continuous expression of interferon regulatory factor 4 sustains CD8+ T cell immunity against tumor. Res (Wash D C). (2023) 6:271. doi: 10.34133/research.0271 PMC10765897 38178902

[203] Harrer DC Bezler V Hartley J Herr W Abken H. IRF4 downregulation improves sensitivity and endurance of CAR T cell functional capacities. Front Immunol. (2023) 14:1185618. doi: 10.3389/fimmu.2023.1185618 37287982 PMC10243527

[204] Longabaugh WJR Zeng W Zhang JA Hosokawa H Jansen CS Li L. Bcl11b and combinatorial resolution of cell fate in the T-cell gene regulatory network. Proc Natl Acad Sci U.S.A. (2017) 114:5800–7. doi: 10.1073/pnas.1610617114 PMC5468679 28584128

[205] Liao R Wu Y Qin L Jiang Z Gou S Zhou L. BCL11B and the NuRD complex cooperatively guard T-cell fate and inhibit OPA1-mediated mitochondrial fusion in T cells. EMBO J. (2023) 42:e113448. doi: 10.15252/embj.2023113448 37737560 PMC10620766

[206] Laiosa CV Stadtfeld M Graf T. Determinants of lymphoid-myeloid lineage diversification. Annu Rev Immunol. (2006) 24:705–38. doi: 10.1146/annurev.immunol.24.021605.090742 16551264

[207] Sottile R Panjwani MK Lau CM Daniyan AF Tanaka K Barker JN. Human cytomegalovirus expands a CD8+ T cell population with loss of BCL11B expression and gain of NK cell identity. Sci Immunol. (2021) 6:eabe6968. doi: 10.1126/sciimmunol.abe6968 34559552 PMC8601152

[208] Jiang Z Qin L Tang Y Liao R Shi J He B. Human induced-T-to-natural killer cells have potent anti-tumour activities. biomark Res. (2022) 10:13. doi: 10.1186/s40364-022-00358-4 35331335 PMC8943975

[209] Fu Q Zheng Y Fang W Zhao Q Zhao P Liu L. RUNX-3-expressing CAR T cells targeting glypican-3 in patients with heavily pretreated advanced hepatocellular carcinoma: a phase I trial. EClinicalMedicine. (2023) 63:102175. doi: 10.1016/j.eclinm.2023.102175 37680942 PMC10480529

[210] Second Affiliated Hospital of Guangzhou Medical University. Induced-T cell like NK cellular immunotherapy for cancers that are lack of MHC-I expression. National Library of Medicine, clinicaltrials.gov (2023). Available at: https://clinicaltrials.gov/study/NCT03882840.

[211] Mognol GP González-Avalos E Ghosh S Spreafico R Gudlur A Rao A. Targeting the NFAT: AP-1 transcriptional complex on DNA with a small-molecule inhibitor. Proc Natl Acad Sci U.S.A. (2019) 116:9959–68. doi: 10.1073/pnas.1820604116 PMC6525529 31019078

[212] Salou M Nicol B Garcia A Laplaud D-A. Involvement of CD8+ T cells in multiple sclerosis. Front Immunol. (2015) 6:604. doi: 10.3389/fimmu.2015.00604 26635816 PMC4659893

[213] Gearty SV Dündar F Zumbo P Espinosa-Carrasco G Shakiba M Sanchez-Rivera FJ. An autoimmune stem-like CD8 T cell population drives type 1 diabetes. Nature. (2022) 602:156–61. doi: 10.1038/s41586-021-04248-x PMC9315050 34847567

[214] Lu H Wang H Yan L Shao H Zhang W Shen H. Overexpression of early T cell differentiation-specific transcription factors transforms the terminally differentiated effector T cells into less differentiated state. Cell Immunol. (2020) 353:104118. doi: 10.1016/j.cellimm.2020.104118 32413598

[215] Roychoudhuri R Clever D Li P Wakabayashi Y Quinn KM Klebanoff CA. BACH2 regulates CD8+ T cell differentiation by controlling access of AP-1 factors to enhancers. Nat Immunol. (2016) 17:851–60. doi: 10.1038/ni.3441 PMC4918801 27158840

[216] Mandelbaum J Bhagat G Tang H Mo T Brahmachary M Shen Q. *BLIMP1* is a tumor suppressor gene frequently disrupted in activated B cell-like diffuse large B cell lymphoma. Cancer Cell. (2010) 18:568–79. doi: 10.1016/j.ccr.2010.10.030 PMC3030476 21156281

[217] Feng Y Pan L Zhang B Huang H Ma H. BATF acts as an oncogene in non−small cell lung cancer. Oncol Lett. (2020) 19:205–10. doi: 10.3892/ol.2019.11075 PMC6924102 31897131

[218] Das AT Tenenbaum L Berkhout B. Tet-on systems for doxycycline-inducible gene expression. Curr Gene Ther. (2016) 16:156–67. doi: 10.2174/1566523216666160524144041 PMC5070417 27216914

[219] Kotter B Engert F Krueger W Roy A Rawashdeh WA Cordes N. Titratable pharmacological regulation of CAR T cells using zinc finger-based transcription factors. Cancers (Basel). (2021) 13:4741. doi: 10.3390/cancers13194741 34638227 PMC8507528

[220] Tousley AM Rotiroti MC Labanieh L Rysavy LW Kim W-J Lareau C. Co-opting signalling molecules enables logic-gated control of CAR T cells. Nature. (2023) 615:507–16. doi: 10.1038/s41586-023-05778-2 PMC10564584 36890224

[221] Choe JH Watchmaker PB Simic MS Gilbert RD Li AW Krasnow NA. SynNotch-CAR T cells overcome challenges of specificity, heterogeneity, and persistence in treating glioblastoma. Sci Transl Med. (2021) 13:eabe7378. doi: 10.1126/scitranslmed.abe7378 33910979 PMC8362330

[222] Hyrenius-Wittsten A Su Y Park M Garcia JM Alavi J Perry N. SynNotch CAR circuits enhance solid tumor recognition and promote persistent antitumor activity in mouse models. Sci Transl Med. (2021) 13:eabd8836. doi: 10.1126/scitranslmed.abd8836 33910981 PMC8594452

[223] Morsut L Roybal KT Xiong X Gordley RM Coyle SM Thomson M. Engineering customized cell sensing and response behaviors using synthetic notch receptors. Cell. (2016) 164:780–91. doi: 10.1016/j.cell.2016.01.012 PMC4752866 26830878

[224] Kim S Park CI Lee S Choi HR Kim CH. Reprogramming of IL-12 secretion in the PDCD1 locus improves the anti-tumor activity of NY-ESO-1 TCR-T cells. Front Immunol. (2023) 14:1062365. doi: 10.3389/fimmu.2023.1062365 36793716 PMC9923015

[225] Jan M Scarfò I Larson RC Walker A Schmidts A Guirguis AA. Reversible ON- and OFF-switch chimeric antigen receptors controlled by lenalidomide. Sci Transl Med. (2021) 13:eabb6295. doi: 10.1126/scitranslmed.abb6295 33408186 PMC8045771

[226] Kim MS Bhargava HK Shavey GE Lim WA El-Samad H Ng AH. Degron-based bioPROTACs for controlling signaling in CAR T cells. ACS Synth Biol. (2024) 13:2313–27. doi: 10.1021/acssynbio.4c00109 PMC11334183 38991546

[227] Mass E Nimmerjahn F Kierdorf K Schlitzer A. Tissue-specific macrophages: how they develop and choreograph tissue biology. Nat Rev Immunol. (2023) 23:563–79. doi: 10.1038/s41577-023-00848-y PMC10017071 36922638

[228] Mazzurana L Czarnewski P Jonsson V Wigge L Ringnér M Williams T. Tissue-specific transcriptional imprinting and heterogeneity in human innate lymphoid cells revealed by full-length single-cell RNA-sequencing. Cell Res. (2021) 31:554–568. doi: 10.1038/s41422-020-00445-x PMC8089104 33420427

[229] Crowl JT Heeg M Ferry A Milner JJ Omilusik KD Toma C. Tissue-resident memory CD8+ T cells possess unique transcriptional, epigenetic and functional adaptations to different tissue environments. Nat Immunol. (2022) 23:1121–31. doi: 10.1038/s41590-022-01229-8 PMC10041538 35761084

[230] Betof Warner A Corrie PG Hamid O. Tumor-infiltrating lymphocyte therapy in melanoma: facts to the future. Clin Cancer Res. (2023) 29:1835–54. doi: 10.1158/1078-0432.CCR-22-1922 PMC10183807 36485001

[231] Rohaan MW Borch TH van den Berg JH Met Ö Kessels R Geukes Foppen MH. Tumor-infiltrating lymphocyte therapy or ipilimumab in advanced melanoma. N Engl J Med. (2022) 387:2113–25. doi: 10.1056/NEJMoa2210233 36477031

[232] Phillips C. Lifileucel first cellular therapy approved for cancer - NCI (2024). Available online at: https://www.cancer.gov/news-events/cancer-currents-blog/2024/fda-amtagvi-til-therapy-melanoma (Accessed March 20, 2024).

[233] Andreatta M Corria-Osorio J Müller S Cubas R Coukos G Carmona SJ. Interpretation of T cell states from single-cell transcriptomics data using reference atlases. Nat Commun. (2021) 12:2965. doi: 10.1038/s41467-021-23324-4 34017005 PMC8137700

[234] Caushi JX Zhang J Ji Z Vaghasia A Zhang B Hsiue EH-C. Transcriptional programs of neoantigen-specific TIL in anti-PD-1-treated lung cancers. Nature. (2021) 596:126–32. doi: 10.1038/s41586-021-03752-4 PMC8338555 34290408

[235] Klichinsky M Ruella M Shestova O Lu XM Best A Zeeman M. Human chimeric antigen receptor macrophages for cancer immunotherapy. Nat Biotechnol. (2020) 38:947–53. doi: 10.1038/s41587-020-0462-y PMC7883632 32361713

[236] Barnes SA Trew I de Jong E Foley B. Making a killer: selecting the optimal natural killer cells for improved immunotherapies. Front Immunol. (2021) 12:765705. doi: 10.3389/fimmu.2021.765705 34777383 PMC8578927

[237] Pan K Farrukh H Chittepu VCSR Xu H Pan C Zhu Z. CAR race to cancer immunotherapy: from CAR T, CAR NK to CAR macrophage therapy. J Exp Clin Cancer Res. (2022) 41:119. doi: 10.1186/s13046-022-02327-z 35361234 PMC8969382

[238] Deng Z Loyher P-L Lazarov T Li L Shen Z Bhinder B. The nuclear factor ID3 endows macrophages with a potent anti-tumour activity. Nature. (2024) 626:864–73. doi: 10.1038/s41586-023-06950-4 PMC10881399 38326607

[239] Lambert N Moussaoui ME Baron F Maquet P Darcis G. Virus-specific T-cell therapy for viral infections of the central nervous system: A review. Viruses. (2023) 15(7):1510. doi: 10.3390/v15071510 37515196 PMC10383098

[240] Raffin C Vo LT Bluestone JA. Treg cell-based therapies: challenges and perspectives. Nat Rev Immunol. (2020) 20:158–72. doi: 10.1038/s41577-019-0232-6 PMC7814338 31811270

[241] Deo SS Gottlieb DJ. Adoptive T-cell therapy for fungal infections in haematology patients. Clin Transl Immunol. (2015) 4:e40. doi: 10.1038/cti.2015.16 PMC4558438 26366286

[242] Mao Y Liao Q Zhu Y Bi M Zou J Zheng N. Efficacy and safety of novel multifunctional M10 CAR-T cells in HIV-1-infected patients: a phase I, multicenter, single-arm, open-label study. Cell Discovery. (2024) 10:1–16. doi: 10.1038/s41421-024-00658-z 38740803 PMC11091177

[243] Müller F Taubmann J Bucci L Wilhelm A Bergmann C Völkl S. CD19 CAR T-cell therapy in autoimmune disease — A case series with follow-up. New Engl J Med. (2024) 390:687–700. doi: 10.1056/NEJMoa2308917 38381673

[244] Maude Shannon L Laetsch Theodore W Buechner J Rives S Boyer M Bittencourt H. Tisagenlecleucel in children and young adults with B-cell lymphoblastic leukemia. N Engl J Med. (2018) 378:439–48. doi: 10.1056/NEJMoa1709866 PMC5996391 29385370

[245] Fowler NH Dickinson M Dreyling M Martinez-Lopez J Kolstad A Butler J. Tisagenlecleucel in adult relapsed or refractory follicular lymphoma: the phase 2 ELARA trial. Nat Med. (2022) 28:325–32. doi: 10.1038/s41591-021-01622-0 34921238

[246] Schuster SJ Bishop MR Tam CS Waller EK Borchmann P McGuirk JP. Tisagenlecleucel in adult relapsed or refractory diffuse large B-cell lymphoma. N Engl J Med. (2019) 380:45–56. doi: 10.1056/NEJMoa1804980 30501490

[247] Locke FL Miklos DB Jacobson CA Perales M-A Kersten M-J Oluwole OO. Axicabtagene ciloleucel as second-line therapy for large B-cell lymphoma. N Engl J Med. (2022) 386:640–54. doi: 10.1056/NEJMoa2116133 34891224

[248] Jacobson CA Chavez JC Sehgal AR William BM Munoz J Salles G. Axicabtagene ciloleucel in relapsed or refractory indolent non-Hodgkin lymphoma (ZUMA-5): a single-arm, multicentre, phase 2 trial. Lancet Oncol. (2022) 23:91–103. doi: 10.1016/S1470-2045(21)00591-X 34895487

[249] Shah BD Ghobadi A Oluwole OO Logan AC Boissel N Cassaday RD. KTE-X19 for relapsed or refractory adult B-cell acute lymphoblastic leukaemia: phase 2 results of the single-arm, open-label, multicentre ZUMA-3 study. Lancet. (2021) 398:491–502. doi: 10.1016/S0140-6736(21)01222-8 34097852 PMC11613962

[250] Wang M Munoz J Goy A Locke FL Jacobson CA Hill BT. KTE-X19 CAR T-cell therapy in relapsed or refractory mantle-cell lymphoma. N Engl J Med. (2020) 382:1331–42. doi: 10.1056/NEJMoa1914347 PMC7731441 32242358

[251] Abramson JS Palomba ML Gordon LI Lunning MA Wang M Arnason J. Lisocabtagene maraleucel for patients with relapsed or refractory large B-cell lymphomas (TRANSCEND NHL 001): a multicentre seamless design study. Lancet. (2020) 396:839–52. doi: 10.1016/S0140-6736(20)31366-0 32888407

[252] Munshi NC Anderson LD Shah N Madduri D Berdeja J Lonial S. Idecabtagene vicleucel in relapsed and refractory multiple myeloma. N Engl J Med. (2021) 384:705–16. doi: 10.1056/NEJMoa2024850 33626253

[253] Berdeja JG Madduri D Usmani SZ Jakubowiak A Agha M Cohen AD. Ciltacabtagene autoleucel, a B-cell maturation antigen-directed chimeric antigen receptor T-cell therapy in patients with relapsed or refractory multiple myeloma (CARTITUDE-1): a phase 1b/2 open-label study. Lancet. (2021) 398:314–24. doi: 10.1016/S0140-6736(21)00933-8 34175021

